# Discovery of Novel Glycosidase-Derived Cell-Penetrating Peptides Encoded by Human Gut Commensals

**DOI:** 10.1021/acssynbio.6c00031

**Published:** 2026-07-09

**Authors:** Sarah E. Post, Hannah S. Ceisler, Rohit G. Lal, Adarsh Singh, Maya A. Deen, Locke E. Bonomo, Lille M. Cunic, Ilana L. Brito

**Affiliations:** Meinig School for Biomedical Engineering, 5922 Cornell University, Ithaca 14850, New York, United States

**Keywords:** cell-penetrating peptide, gut microbiome, functional metagenomics, bacterial display

## Abstract

Intracellular delivery of therapeutics remains a major challenge for modern medicine. To enhance intracellular uptake, therapeutics can be delivered with carrier proteins possessing an inherent cell-penetrating activity. There is an increasing need for new cell-penetrating carriers with diverse biophysical properties and mechanisms of action to transport a wide range of therapeutic cargo. As many cell-penetrating proteins and peptides derive from natural proteins, we sought to mine a previously unexplored community, the human gut microbiome, for cell-penetrating sequences. Here, we performed a high-throughput functional metagenomic screen to identify cell-penetrating protein fragments from the human gut microbiome. We identified protein fragments encoded within glycosidase enzymes from members of the Bacteroidetes phylum that mediate internalization into human cell lines when displayed on the surface of nonpathogenic, noninvasive *Escherichia coli*. We investigate one fragment, dubbed Gh_112, that adheres to human fibronectin, activates multiple endocytic pathways, and specifically promotes uptake of *E. coli* into multiple cancerous epithelial cell lines rather than healthy epithelial tissue *in vitro.* Overall, this work demonstrates that the human gut microbiome is a source of cell-penetrating sequences and expands the known repertoire of cell-penetrating carrier systems.

## Graphical abstract

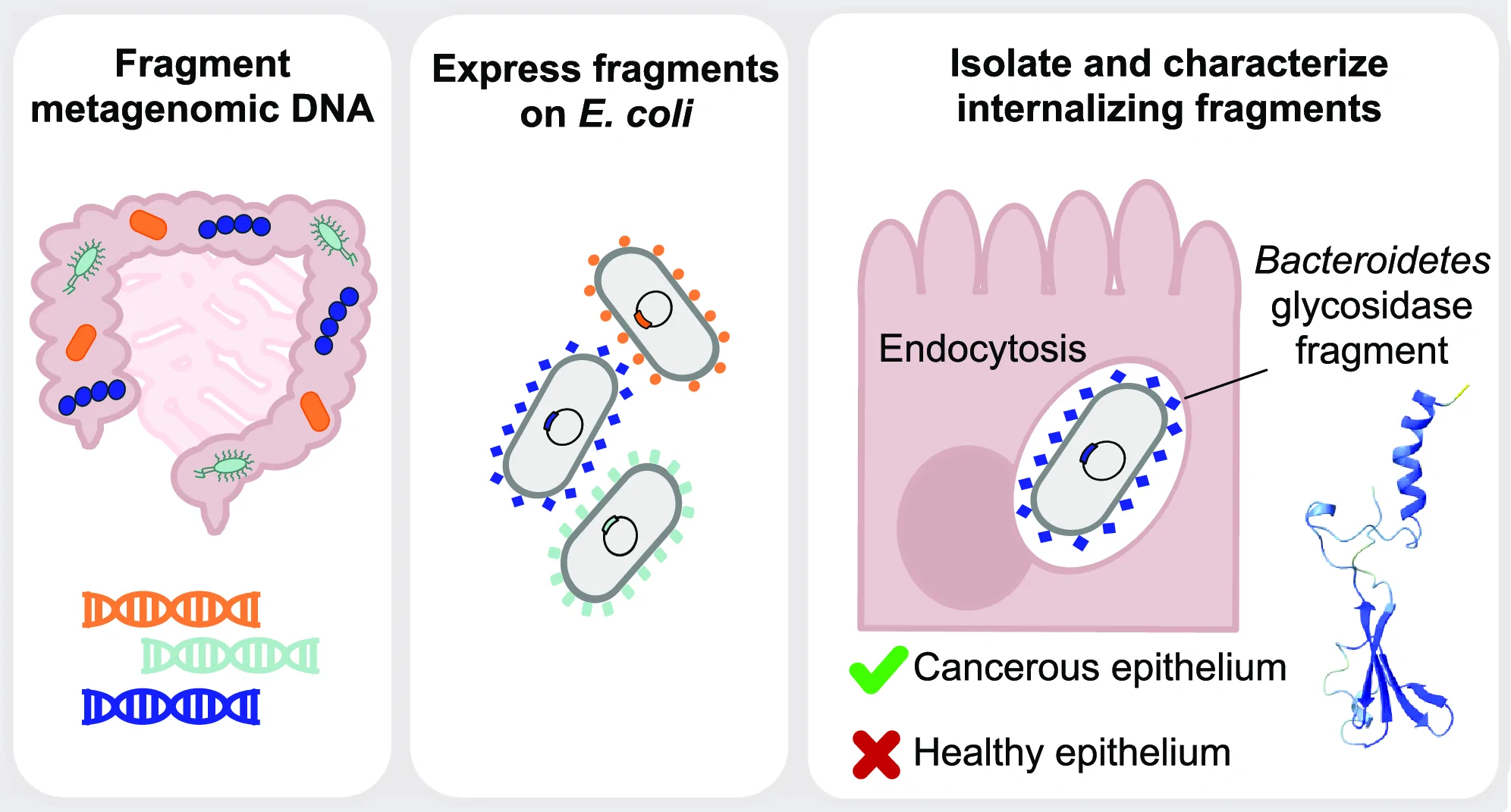

## Introduction

Intracellular delivery of therapeutics remains a major challenge in drug development. Many important therapeutic macromolecules, such as DNA, RNA, and antibodies, are inherently membrane-impermeable and therefore limited in efficacy. To improve intracellular uptake of these therapeutic molecules, development of intracellular drug delivery systems with diverse biophysical properties, mechanisms of action, and tissue specificities is required.

Cell-penetrating peptides (CPPs) are short, charged peptide sequences naturally capable of translocating across the cell membrane and have been widely investigated as drug delivery vehicles.^1^ The CPP charge facilitates interactions with the cell membrane triggering internalization either by endocytosis or direct translocation into the cytosol via membrane pore formation.^2,3^ As most drug targets are located in the cytosol, CPPs are attractive drug carriers, as they can bypass the need for endosomal escape to reach cytosolic targets. Several CPPs capable of cytosolic translocation derive from the proteins of pathogenic microorganisms including the TAT peptide from human immunodeficiency virus.^4^ Despite these advantages, CPPs lack cell-type specificity and are limited in cargo size, or their efficacy is impacted by biophysical properties of the conjugated cargo.^5,6^

Alternatively, pathogenic bacteria produce proteins that promote invasion into the mammalian cell by binding to membrane receptors and triggering entry in a cell-type specific manner. These pathogen-derived cell-penetrating proteins can be similarly functionalized on nanoparticles or attached to nonpathogenic bacteria to create engineered bacterial delivery systems.^7–11^ Bacterial delivery systems have emerged as a promising tool for tumor-targeted delivery as *Escherichia coli* (*E. coli*) and *Salmonella* naturally colonize the hypoxic tumor microenvironment and can be engineered to produce therapeutic payloads upon delivery to the target site.^12,13^ Recently, attenuated *Salmonella* strains were engineered for intracellular delivery of apoptotic peptides or oncolytic viruses to solid tumors.^14,15^ As these delivery systems derive from pathogenic sources, immunogenicity and biosafety remain a concern.

Here, we screen the human gut microbiome, a genetically diverse community of commensal microbes, for CPPs with unique properties and cell-penetrating mechanisms. The human gut microbiome contains millions of uncharacterized protein sequences from diverse bacterial species but, to our knowledge, has not been screened for cell-penetrating sequences. To screen a library of microbiome-derived sequences simultaneously, we leverage functional metagenomics, a culture-independent approach that enables high-throughput screening of microbial gene products for a desired biological activity. In this study, we express protein fragments from a human gut metagenome on the surface of noninvasive, nonpathogenic *Escherichia coli* and screen the resulting bacterial display library for the ability to internalize into human cell lines.

With this library-scale screen, we identify two 100-amino acid protein fragments from commensal-derived glycosidases that drive uptake of *E. coli* into human epithelial tissue when displayed on the bacterial surface. We further investigate one fragment, dubbed Gh_112, which activates multiple endocytic processes to internalize into cancerous human cell lines but not healthy primary tissue *in vitro*. Overall, in this study, we identify a CPP derived from the human gut microbiome and expand the known repertoire of cell-penetrating systems with cell-selective properties.

## Results

### Library-Scale Internalization Assay Enables Rapid Discovery of Microbiome-Derived Cell-Penetrating Sequences

We employed a high-throughput functional metagenomic screen based on bacterial surface display technology and library-scale gentamicin protection assays to identify protein fragments with cell-penetrating activity.^16^ A surface-displayed metagenomic library was constructed from the stool of a healthy human donor with no history of antibiotic usage in the past 6 months (Figure S1A,B). Briefly, metagenomic DNA was extracted, sheared to ∼500 bp fragments, and inserted into a bacterial surface display vector pAIDA1. The fragments were expressed on the extracellular-exposed N-terminus of the transmembrane protein “Autotransporter involved in diffuse adherence” (AIDA-I) on the surface of BL21 *E. coli*, a nonpathogenic, noninvasive *E. coli* strain (Figure S2A,B). While wild-type AIDA-I functions as an adhesin, in the AIDA-I display system, the adhesive passenger domain is replaced by the sequence of interest.^17,18^ Culture of the metagenomic display library on gentamicin plates prior to biopanning confirmed that the library was gentamicin sensitive at concentrations used in this assay, as no colonies formed from the plated library.

The metagenomic display library was subjected to three iterative rounds of a library-scale gentamicin protection assay (Figure 1A). As the library derives from fecal bacteria, we screened for internalization into differentiated Caco-2, a colorectal cancer cell line that spontaneously differentiates into an intestinal enterocyte-like phenotype. Caco-2 differentiation was confirmed by qPCR for expression of the sucrase-isomaltase gene (Figure S3).^19^ The metagenomic display library was coincubated with Caco-2 in biological triplicate, allowing bacteria displaying cell-penetrating sequences to translocate into the mammalian cell interior. The cells were then treated with the antibiotic gentamicin, which is impermeable to the mammalian cell membrane, to kill extracellular *E. coli* while intracellular bacteria are protected from antibiotic exposure. The gentamicin is removed, mammalian cells lysed to release intracellular *E. coli*, and surviving bacteria are recovered and sequenced to identify cell-penetrating clones.^16^ The recovered *E. coli* are expanded and subjected to two additional rounds of the gentamicin protection assay, or “biopanned”, to further enrich for cell-penetrating clones.

**Figure 1:**
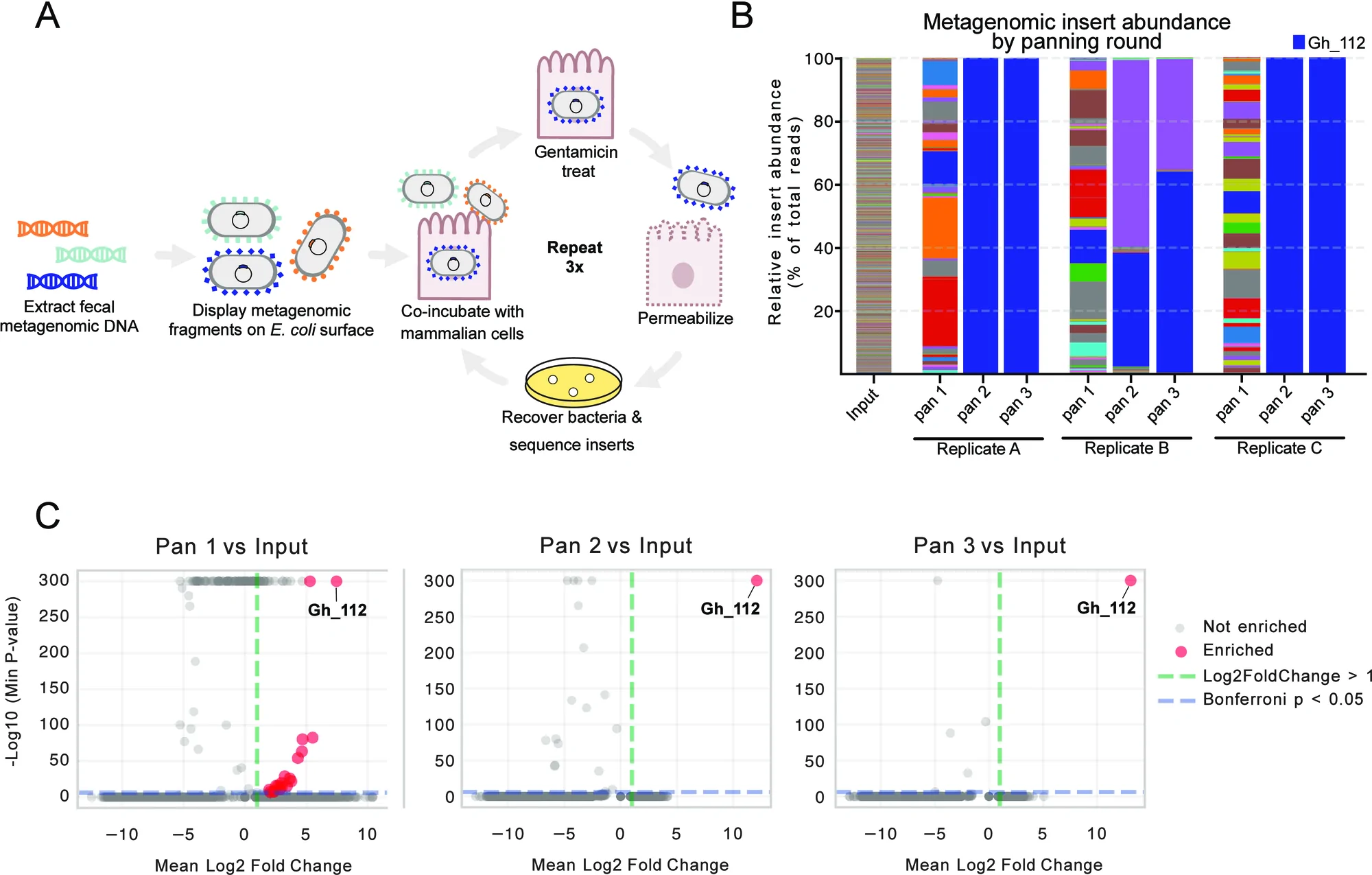
Functional metagenomic internalization assay enriches for a protein fragment from a human gut microbiome. (A) Overview of the high-throughput functional metagenomic internalization assay. (B) Relative read abundances mapping to unique metagenomic inserts from the pre-gentamicin selected “input library” and panning round 1–3 recoveries across three biological replicates. Read abundances calculated as percent of reads mapped to unique insert over total reads within each population. Each individual stack within a bar corresponds to a unique metagenomic insert. (C) Volcano plot of metagenomic insert enrichment from pan 1–pan 3 as compared to the preselected input population. Inserts passing significant enrichment criteria (positive log2FC > 1, Bonferroni-adjusted *p*-value <0.05 in 2+ replicates) indicated in red.

After sequencing, a single clone denoted as “Gh_112” represented the most abundant sequence at 60 to >99% of the population across all three biological replicates by the third panning round and steadily increased in abundance from pan 1 through pan 3 (Figure 1B). To determine statistical enrichment of metagenomic inserts, data was fitted to a generalized Poisson model. Sequences were called as enriched in the selections if they demonstrate a Bonferroni-adjusted *p*-value <0.05 and positive log2FoldChange >1 in at least two replicates (Figure 1C).

### Fecal Metagenomic Selections Identify a Protein Fragment with Cell-Penetrating Activity

The Gh_112 sequence was purchased and cloned into a clean pAIDA-I backbone to compare internalization of *E. coli* displaying these fragments to a panel of control proteins. The cell-penetrating domains 2 and 3 from the *Staphylococcus aureus* “Fibronectin Binding Protein A” (FnBP_A(2–3)), the Caco-2 adhesin “Mammalian adhesion protein A” (MapA) from *Lactobacillus reuteri*, or the AIDA-I display scaffold lacking an insert (“no insert”) served as controls.^20,21^ Surface localization of Gh_112 and controls was confirmed by detection of the Myc tag between the C-terminus of the metagenomic insert and AIDA-I surface anchor on bacteria with intact membranes by flow cytometry (Figure 2A and Figure S4). Gh_112 and all controls successfully displayed on the *E. coli* surface. Importantly, *E. coli* displaying Gh-112 were fully gentamicin susceptible at concentrations used in this assay, supporting that gentamicin survival is likely due to internalization into Caco-2 (Figure S5A–C).

**Figure 2:**
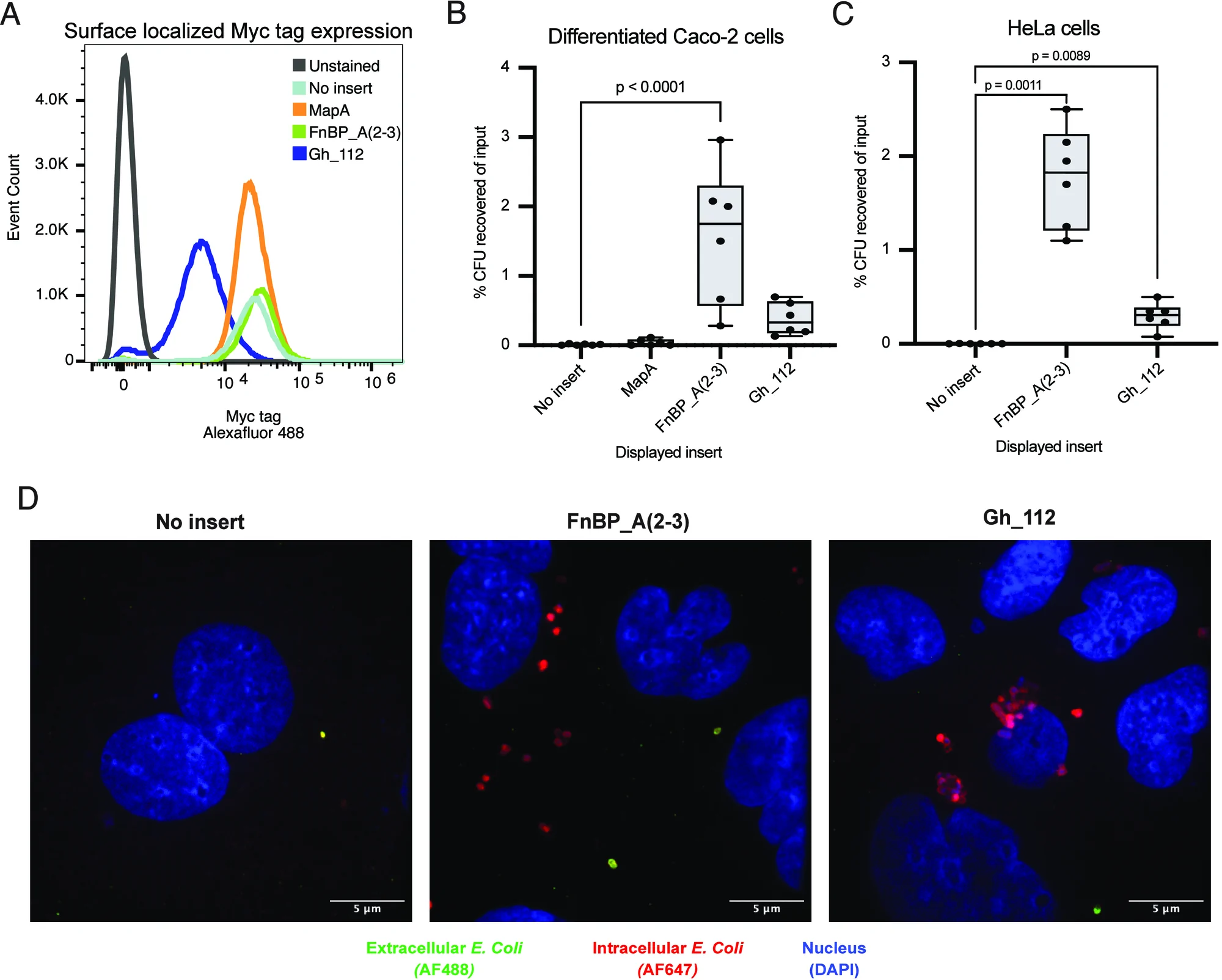
Displayed Gh_112 fragment drives internalization into mammalian cell lines. (A) Surface localization of the AIDA1-displayed proteins Fibronectin Binding Protein A domains 2–3 (FnBP_A(2–3)), Mammalian adhesion protein A (MapA), or Gh_112. Localization is measured by detection of the Myc tag on propidium-iodide negative (intact membrane) bacterial cell population via flow cytometry. (B) Relative recovery of *E. coli* displaying Fibronectin Binding Protein A domains 2–3 (FnBP_A(2–3)), Mammalian adhesion protein A (MapA), Gh_112, or no insert from the pAIDA1 vector into differentiated Caco-2 or (C) HeLa *n* = 3, technical duplicates (six data points per condition shown). Error bars reflect standard deviation, only significant comparisons (*p* < 0.05) indicated with brackets and reported *p*-values on graph as measured via one-way ANOVA with post hoc Dunnett’s multiple comparisons against the “no insert” control. (D) Localization of *E. coli* displaying Gh_112 or controls incubated with differentiated CaCo-2. Extracellular bacteria stained green (Alexa Fluor 488) on intact cells, followed by permeabilization and staining of total bacteria in red (Alexa Fluor 647). Nucleus stained with DAPI. Imaged on Andor spinning disc confocal microscope at 100× magnification.

We then performed a clonal version of the invasion assay by individually incubating bacteria displaying Gh_112 or controls with differentiated Caco-2 at an MOI of 10:1 bacteria/mammalian cells. Relative internalization is calculated as the percentage of colony-forming units (CFUs) surviving gentamicin treatment relative to the input CFU, as determined by colony counting of the input or recovered cells. *E. coli* displaying the fragment Gh_112 and the internalizing control FnBP_A(2–3) survive gentamicin treatment at higher rates than *E. coli* displaying the no insert control or the adhesive protein MapA (Figure 2B).

The pAIDA-I vector contains an N-terminal hexahistidine tag, which, under acidic conditions, can become positively charged and may facilitate interaction with the cell membrane.^22^ To confirm that the histidine residues are not driving Gh_112 internalization, we generated an alternate version of the vector, pAIDA-I_v2, which lacks the N-terminal hexahistidine tag and introduces a flexible serine-glycine linker between the C-terminus of the insert and start of the AIDA-I transmembrane anchor to allow for freer insert folding (Figure S6A). Gh_112 mediated significant internalization into Caco-2 even without histidine residues at levels comparable to the pAIDA-I vector, indicating that the hexahistidine tag is not driving internalization (Figure S6B). Gh_112 and FnBP_A(2–3) similarly promoted bacterial internalization into the human cervical epithelial HeLa cell line, indicating that internalization is not restricted to colonic epithelial Caco-2 cells (Figure 2C). Finally, internalization of Gh_112-displaying *E. coli* was qualitatively confirmed by differential staining to image intracellular and extracellular bacteria.^23^ *E. coli* displaying Gh_112, FnBP_A(2–3), or “no insert” were incubated with Caco-2. The cells were first washed to remove unbound bacteria, fixed with a nonpermeabilizing fixative, and stained with an anti-*E. coli* antibody and in green secondary antibody to label extracellular bacteria. The monolayers are then permeabilized and restained with the same anti-*E. coli* antibody this time labeled with a red secondary to mark all bacteria. While few bacteria were present overall in the “no insert” control wells, we readily observe bacteria displaying Gh_112 and FnBP_A(2–3) stained with the red postpermeabilization fluorophore, confirming intracellular localization (Figure 2D). Notably, we observe only select cells in a field of view uptake bacteria displaying FnBP_A(2–3) or Gh_112. This is consistent with the low uptake rate quantified by the gentamicin protection assay and well-described cell-to-cell heterogeneity in rates of endocytic uptake.^24^

### Native Glycosidase Containing Gh_112 Lacks Internalization Activity on the Surface Display System

Gh_112 originates in-frame from a glycosidase (UniProt A0A1Y3QXY7) of the human gut bacterium *Alistipes onderdonkii* (Figure 3A).^25^ The Gh_112 protein fragment represents a 100-amino acid, 11.2 kDa protein fragment with an estimated isoelectric point of 5.09 and defined secondary structure as predicted by AlphaFold2 (Figure 3B). To determine whether the “parental” glycosidase also demonstrates cell-penetrating activity, we displayed the glycosidase on *E. coli* (Figure 3C) and subjected it to the internalization assay over Caco-2. No internalization of bacteria displaying the glycosidase was detected, suggesting that Gh_112 acts independently of the full enzyme to mediate internalization on the display system (Figure 3D).

**Figure 3:**
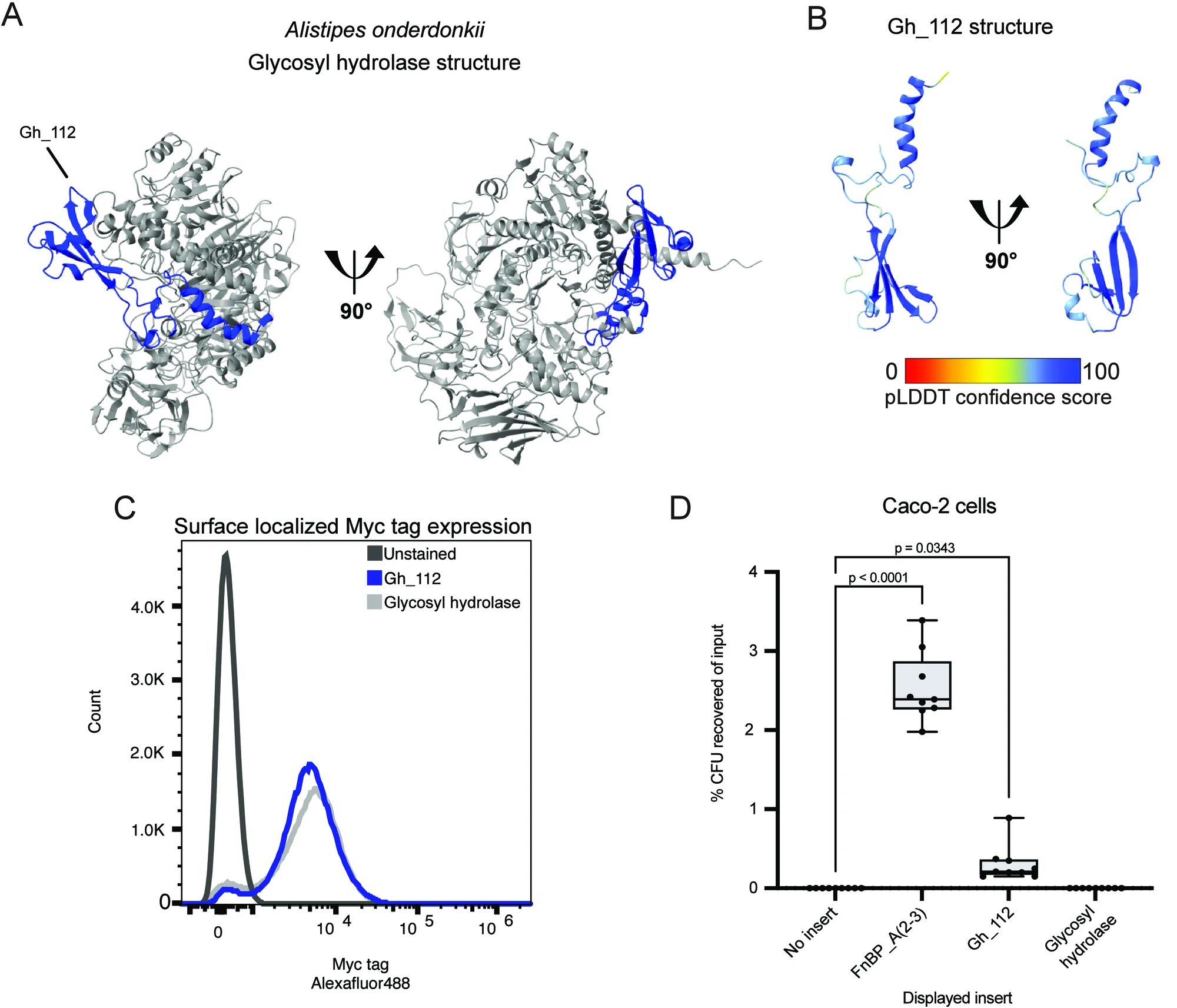
Gh_112 acts independently of the parental glycosyl hydrolase. (A) AlphaFold2-predicted structure of the *Alistipes onderdonkii* glycosyl hydrolase (UniProt A0A1Y3QXY7) with the Gh_112 fragment indicated in blue. (B) AlphaFold2-predicted structure of the 100-amino acid Gh_112 fragment colored by pLDDT confidence score. (C) Surface localization of the glycosyl hydrolase on the *E. coli* surface measured by detection of the Myc tag on propidium iodide-negative (intact membrane) bacterial cell population via flow cytometry. (D) Relative recovery of *E. coli* displaying the Gh_112 fragment versus the mature native glycosyl hydrolase protein following incubation with Caco-2. *n* = 3, technical triplicates (nine data points per condition shown). Error bars reflect standard deviation, only significant comparisons (*p* < 0.05) indicated with brackets and reported *p*-values on graph as measured via one-way ANOVA with posthoc Dunnett’s multiple comparisons against the “no insert” control.

### A Homologous Glycosidase Fragment from *Bacteroides eggerthii* Similarly Mediates *E. coli* Internalization

A BLASTP search of Gh_112 against the curatedMetagenomicData set, a representative database of proteins from over 22,000 sequenced human metagenomes, identified 19 homologous fragments all derived from commensal glycosidases (Figure 4A,B).^26^ Screening of select representative clade members identifies a glycosidase fragment from another member of the Bacteroidetes phylum, *Bacteroides eggerthii*, that similarly promotes *E. coli* internalization on the display system (Figure 4C,D).

**Figure 4:**
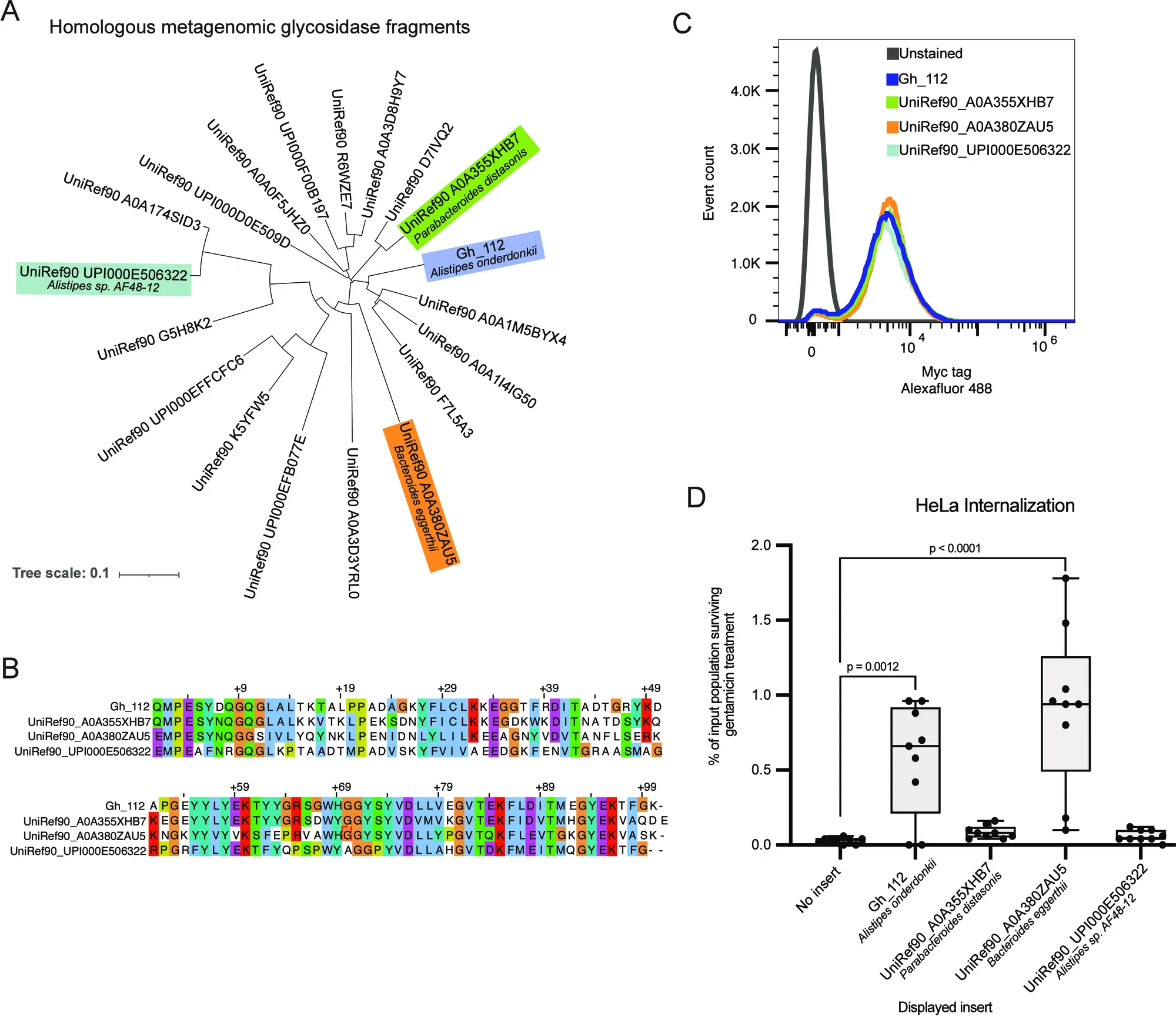
Homologous glycosidase fragment mediates *E. coli* internalization. (A) Phylogenetic tree of protein fragments from human metagenomes with significant homology to Gh_112 as determined by BLASTP search of the curatedMetagenomicData data set. Gh_112 and select fragments tested in the internalization assay indicated by colored boxes. (B) Multiple sequence alignment of Gh_112 with fragments tested in internalization assay aligned with ClustalOmega and colored with default clustal color scheme. (C) Surface localization of the AIDA1-displayed Gh_112 or homologue fragments measured by detection of the Myc tag on propidium-iodide negative (intact membrane) bacterial cell population via flow cytometry. (D) Relative recovery of *E. coli* displaying Gh_112 or homologous fragments into HeLa cells *n* = 3, technical triplicates (nine data points per condition shown). Error bars reflect standard deviation only significant comparisons (*p* < 0.05) indicated with brackets and reported *p*-values on graph as measured via one-way ANOVA with post hoc Dunnett’s multiple comparisons against the “no insert” control.

### Gh_112 Represents a Novel Cell-Penetrating Protein Fragment

To determine whether Gh_112 contains any known cell-penetrating sequences, the Gh_112 sequence was queried for exact matches against all natural peptide sequences present in CPPSite3, a comprehensive database of over 4000 experimentally verified cell-penetrating peptides.^27^ A four-amino-acid peptide “LCLK” with known cell-penetrating activity is present within Gh_112 at amino acid positions 29–32.^28^ We mutated the corresponding residues within the Gh_112 sequence from “LCLK” to four alanine residues “Gh_112_29_(AAAA)_32_” and confirmed surface localization of the mutated sequence (Figure 5A,B). After subjecting both fragments to the internalization assay, Gh_112-mediated *E. coli* internalization even in the absence of “LCLK”, although at a lower rate, suggesting that Gh_112 possesses cell-penetrating activity independent of the known CPP sequence (Figure 5C).

**Figure 5:**
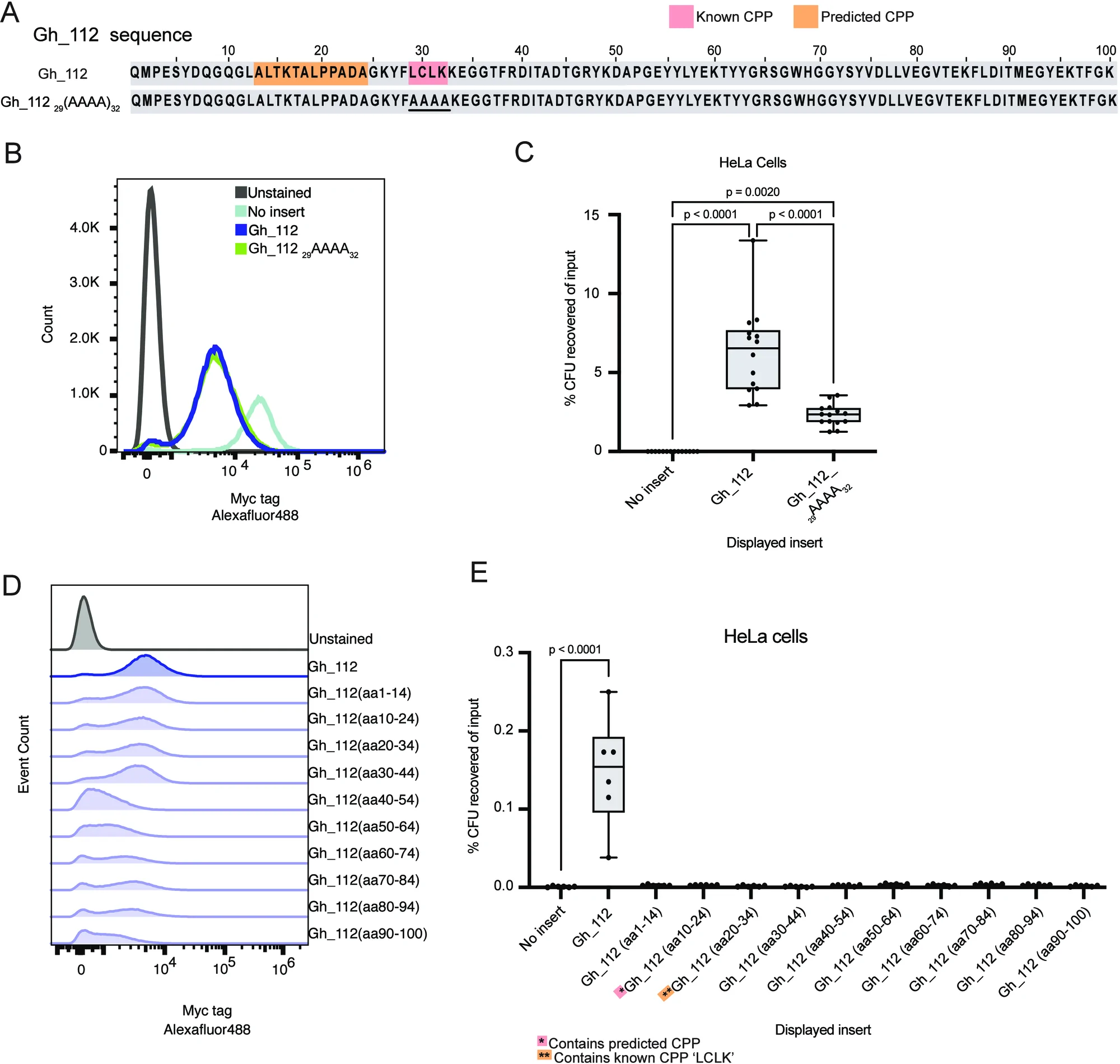
Cell-penetrating function of Gh_112 is not dependent on known CPPs. (A) Amino acid sequence of Gh_112 with the known cell-penetrating peptide sequence “LCLK” at amino acid positions 29–32 highlighted in red and predicted CPP at positions 13–24 indicated in orange. Aligned sequence Gh_112_29­(AAAA)­32 indicates the Gh_112 fragment with the cell-penetrating peptide at residues 29–32 mutated to four alanines. (B) Surface localization of Gh_112_29­(AAAA)­32 as compared to wild-type Gh_112. Localization is measured by detection of the Myc tag on the propidium-iodide-negative (intact membrane) bacterial cell population via flow cytometry. (C) Relative recovery of *E. coli* displaying the wild-type Gh_112 fragment or Gh_112_29­(AAAA)­32 following incubation with HeLa cells. *n* = 3, technical triplicates (nine data points per condition shown). Error bars reflect standard deviation, only significant comparisons (*p* < 0.05) indicated with brackets and reported *p*-values on graph as measured via one-way ANOVA with post hoc Tukey’s multiple comparisons. (D) Surface localization of Gh_112 fragments on the *E. coli* surface as measured by flow cytometry. (E) Relative recovery of *E. coli* displaying Gh_112 or 14aa tiled fragments of Gh_112 into HeLa cells. *n* = 3, technical triplicates (nine data points per condition shown). Error bars reflect standard deviation, only significant comparisons (*p* < 0.05) indicated with brackets and reported *p*-values on graph as measured via one-way ANOVA with post hoc Dunnett’s multiple comparisons against the “no insert” control.

We next leveraged the CPP-prediction algorithm CellPPD to screen Gh_112 for sequences with CPP-like properties within the larger 100-amino acid sequence.^29,30^ A 10-amino-acid-long peptide within Gh_112 at amino acid positions 13 to 24 was predicted to contain CPP-like properties (Figure 5A). To screen this peptide and additional fragments for cell-penetrating activity, Gh_112 was fragmented into 10–14 amino acid peptides with four amino acids of overlap. Despite displaying on the bacterial surface, none of the fragments, including Fragment 2, which contained the predicted CPP or Fragment 3 containing “LCLK”, mediated internalization of bacteria into HeLa cells (Figure 5D,E). This suggests that the ability of Gh_112 to facilitate *E. coli* internalization is driven by a larger structure or sequence within the 100-amino acid fragment. We attempted to display Gh_112 in two 50-amino acid halves on the display system to query larger sequences for internalization, but these halves did not display on the cell surface (Figure S7).

### Gh_112 Engages Multiple Endocytic Pathways to Cross the Host Cell Membrane

To identify specific pathways important for uptake of Gh_112, HeLa cells were incubated at 4 °C to halt active endocytosis or with a panel of pharmacological endocytosis inhibitors to block specific endocytic pathways.^31,32^ Pharmacological inhibitors did not significantly impact cell viability across treatments (Figure S8). Gh_112 uptake on bacteria is driven by active endocytosis, as evidenced by a complete inhibition of invasion at 4 °C (Figure 6A). Specifically, inhibition of clathrin-mediated endocytosis (chlorpromazine), lipid raft formation (methyl-*B*-cyclodextrin), and actin rearrangement (cytochalasin D) resulted in significant reductions of Gh_112 internalization (Figure 6A). This indicates that Gh_112 leverages multiple pathways to promote endocytosis into the cell. Furthermore, when Gh_112-displaying bacteria are labeled with pHRodo, a dye that selectively fluoresces in acidic environments, we detect cells in low-pH compartments consistent with endocytic uptake (Figure 6B). Interestingly, the involvement of multiple pathways suggests engagement of additional receptors, supporting our findings that “LCLK” is not solely responsible for intracellular entry.

**Figure 6:**
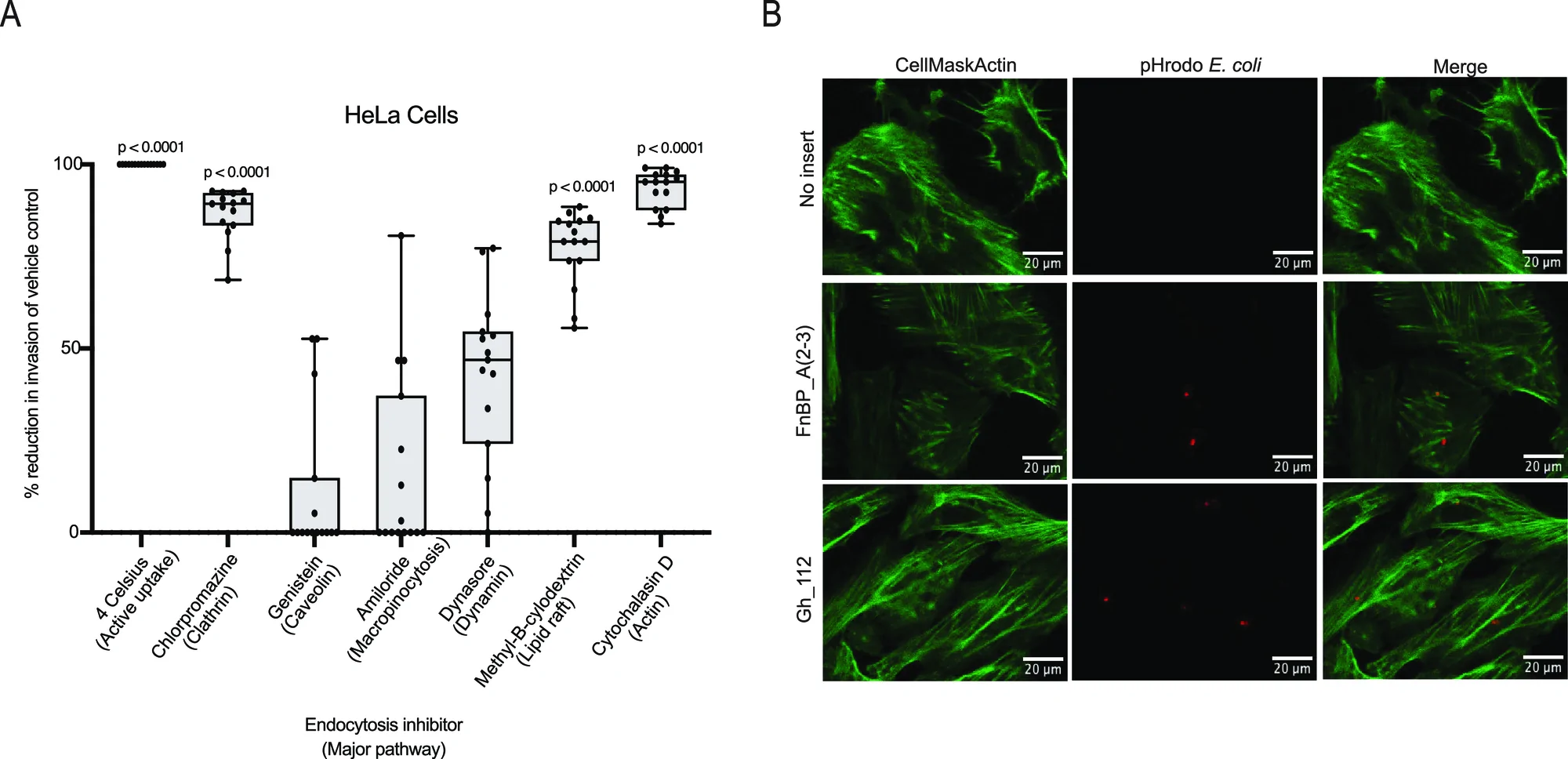
Multiple endocytic pathways contribute to Gh_112 internalization. (A) Relative recovery of Gh_112-displaying *E. coli* after incubation with HeLa cells at 4 °C or the presence of chemical endocytosis inhibitors. Reported as percent reduction in recovery versus vehicle control. *n* = 3, technical triplicates (nine data points per condition shown). *p*-values reflect Student’s *t* test of relative bacterial recovery with inhibitor treatment versus vehicle control only significant comparisons (*p* < 0.05) shown. Error bars indicate standard deviation. (B) Imaging of *E. coli* displaying indicated insert in low-pH compartments following incubation with HeLa cells. *E. coli* labeled with pHRodo Red and the HeLa cell body labeled with CellMask Actin green. Imaged on a Zeiss LSM880.

### Gh_112-Displaying Bacteria Adhere to Human Fibronectin

As Gh_112 leverages multiple mechanisms of endocytosis to internalize into human cell lines, we next investigated how Gh_112 may interact with the mammalian cell surface. FnBP_A(2–3) triggers actin rearrangement by indirect engagement of the alpha-1 beta-5 integrin receptor through fibronectin binding.^20^ As we demonstrated that Gh_112 triggers actin rearrangement, we tested whether Gh_112 can similarly interact with human fibronectin. *E. coli* displaying Gh_112, FnBP_A(2–3), or no insert were stained with SYBR-Gold and incubated on human fibronectin-coated plates. Both FnBP_A(2–3) and Gh_112-displaying bacteria adhered to human fibronectin (Figure 7A,B). Interestingly, we could find no significant structural homology, sequence homology, or motif presence in Gh_112 resembling fibronectin binding sequences or other bacterial invasins.

**Figure 7:**
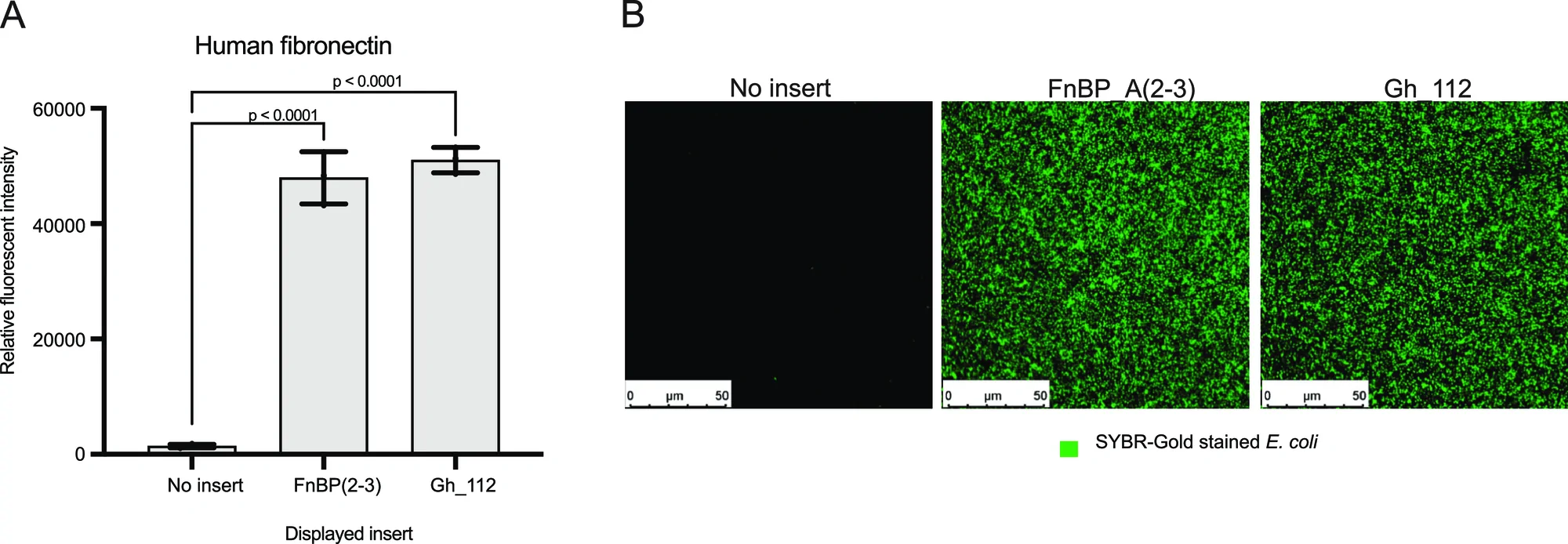
Displayed Gh_112 fragment adheres to human fibronectin. (A) Adhesion of *E. coli* displaying Gh_112 versus FnBP_A(2–3) or “no insert” controls to human fibronectin coated plates. Measured as relative fluorescence intensity of SYBR-Gold stained bacteria on human fibronectin coated plates. *n* = 3, technical triplicates (nine data points per condition shown). Error bars reflect standard deviation, only significant comparisons (*p* < 0.05) indicated with brackets and reported *p*-values on graph as measured via one-way ANOVA with Dunnett’s multiple comparisons versus “no insert” control. (B) Imaging of adherent SYBR-Gold labeled *E. coli* displaying FnBP_A(2–3), Gh_112, or no insert on human fibronectin-coated plates.

### Gh_112 Selectively Promotes Bacterial Uptake into Cancerous, but Not Healthy, Epithelial Cell Lines *In Vitro*

To test whether Gh_112 internalization is specific to cancerous cell lines, Gh_112-displaying bacteria were introduced into the colorectal cancer cell line CaCo-2 or primary colonic epithelial cells (HCoEPiC) isolated from a healthy donor. On the bacterial display system, Gh_112 and FnBP_A(2–3) internalize into undifferentiated and differentiated CaCo-2 cell lines but not primary colonic epithelial cells (Figure 8A). Similarly, Gh_112 readily internalized into immortalized cervical HeLa cells but not primary cervical epithelial tissue (Figure 8B). In contrast, *E. coli* displaying FnBP_A(2–3) had detectable internalization into both cancerous and healthy primary cervical epithelial cells at low levels under the conditions tested.

**Figure 8:**
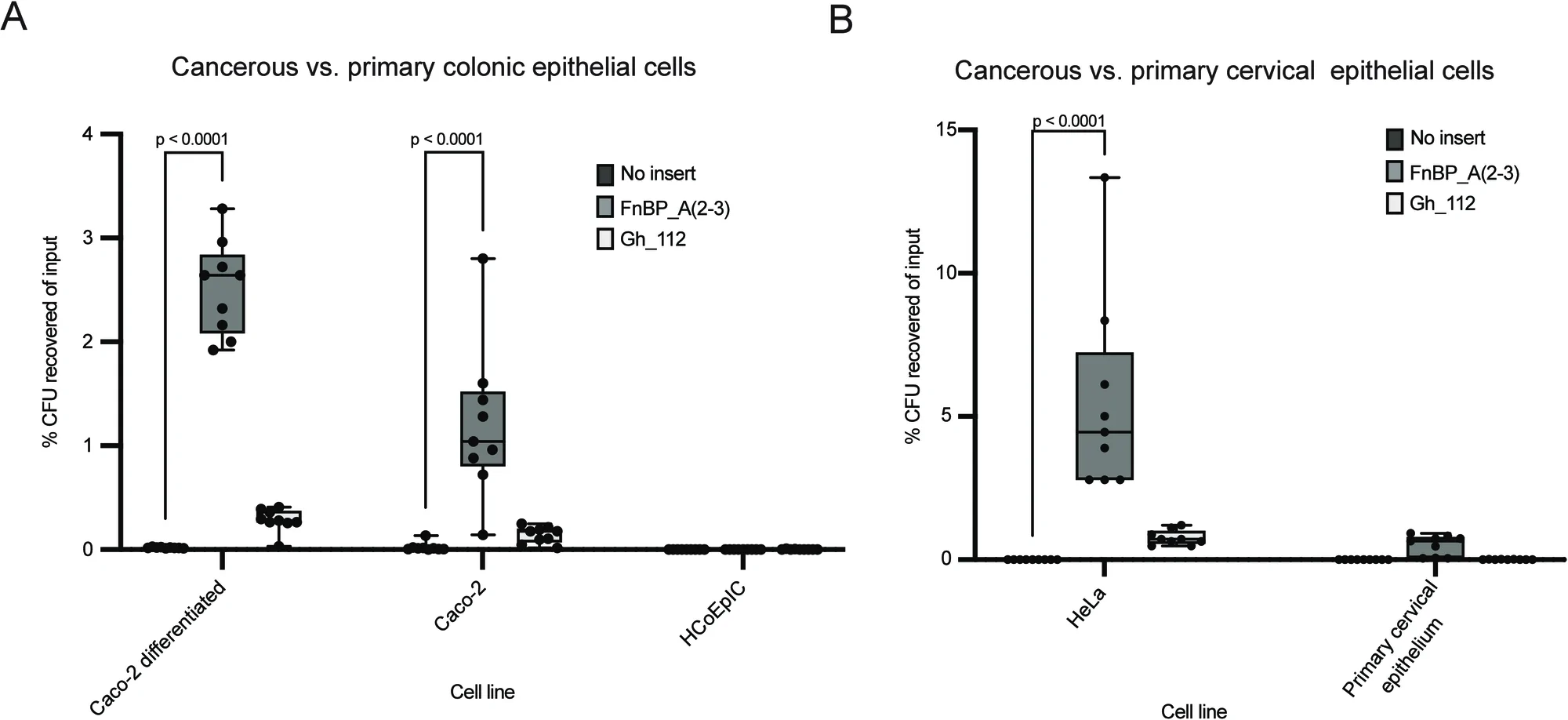
Gh_112 and FnBP_A(2–3) show preferential uptake into cancerous epithelial tissue. (A) Relative recoveries of *E. coli* displaying Gh_112, FnBP_A(2–3), or “no insert” control following incubation with primary human colonic epithelial cells (HCoEpIC), Caco-2, or differentiated Caco-2 (*n* = 3). (B) Relative recoveries of *E. coli* displaying Gh_112, FnBP_A(2–3), or “no insert” following incubation with primary cervical epithelial cells versus HeLa *n* = 3, technical triplicates (nine data points per condition shown). Error bars reflect standard deviation, only significant comparisons (*p* < 0.05) indicated with brackets and reported *p*-values on graph as measured via one-way ANOVA with post hoc Dunnett’s multiple comparisons versus “no insert” control for each cell type.

## Discussion

In this study, we leveraged bacterial surface display technology and a gentamicin protection assay to perform a high-throughput functional metagenomic screen to identify cell-penetrating protein fragments within the human gut bacteria. We identify two 100-amino acid protein fragments within glycosidases encoded by members of the Bacteroidetes phylum, *Alistipes onderdonkii* and *Bacteroides eggerthii*, that drive internalization of large bacterial cargo into mammalian cells. We find that Gh_112 uses multiple endocytic mechanisms to promote uptake of whole, nonpathogenic, noninvasive *E. coli* into human intestinal epithelial cells. In the bacterial display system, Gh_112 mediated preferential internalization into cancer-derived epithelial cell lines rather than healthy tissue. These results demonstrate that a human gut bacterium encodes a cell-penetrating sequence and that the human gut microbiome is a potential source for new CPPs.

Glycosidases belong to a class of carbohydrate-active enzymes (CAZymes) that are widely prevalent among members of the Bacteroidetes phylum.^33,34^ These glycosidases enable carbohydrate utilization by gut bacteria and contribute to the colonization in the intestine. As glycosidases bind to and cleave carbohydrates, it is possible that Gh_112 retains carbohydrate binding function and engages with glycosylation or sugar moieties of cell surface glycoproteins. In fact, *Bacteroides thetaiotaomicron*, another member of the Bacteroidetes phylum, is known to interact with and metabolize glycan groups from host mucins.^35^ Glycan binding is also a known adhesion mechanism of the bacterium *Fusobacterium nucleatum* in the gut, where the bacterial Fap2 protein binds to a polysaccharide moiety overexpressed on cancerous cells, leading to selective colonization of colorectal tumors.^36,37^ To determine whether Gh_112 leverages glycan binding to associate with human cells will require deeper characterization of cell binding mechanisms.

To our knowledge, *Alistipes onderdonkii* and *Bacteroides eggerthii* do not enter human cells, nor is there evidence of glycosidases possessing cell-penetrating properties. Instead, we hypothesize that Gh_112 is a cryptic peptide, where peptide function arises only after release from the larger protein structure. This is consistent with our observation that the native glycosidase protein does not mediate internalization in the display system. Previous studies have demonstrated that the human gut harbors cryptic peptides and small open reading frames that function as immunomodulatory agents or antimicrobial peptides, but to our knowledge, cryptic CPPs have not been identified in the gut microbiome^38,39^ If Gh_112 is naturally released from the glycosidase in the gut, then it could act similarly as an immunomodulatory agent at the epithelial interface. However, to determine the effects of Gh_112 on cell function, further work is needed to characterize interactions with the mammalian cell off the surface display system.

While Gh_112 release from the larger glycosidase is possibly a non-natural phenomenon identified by artificially truncating the glycosidase, the Gh_112 fragment may be exposed in the gut if the glycosidase is released during cell death and then undergoes proteolysis. To understand the impacts of Gh_112 on the host cell biology and a possible physiological role in the gut, further protein engineering efforts are required to purify stable Gh_112. This will allow the study of Gh_112 interaction with human cells independently of the surface display system.

On the surface display system, temperature and pharmacological inhibition of active cell entry mechanisms reveal that Gh_112 entry is dependent on multiple endocytic processes including clathrin-mediated endocytosis, lipid raft formation, and actin rearrangement. The dispensable “LCLK” peptide within Gh_112 is known to use clathrin-mediated endocytosis as the primary route of entry and likely contributes to the multiple endocytic mechanisms triggered by Gh_112. Activation of endocytosis indicates Gh_112 enters human cells by receptor-mediated mechanisms rather than direct membrane translocation. This is consistent with the ability of Gh_112 to transport an entire bacterial cell across the cell membrane as membrane pores formed by CPPs are only ∼2 nm in diameter, too small to allow passive diffusion of whole bacteria. However, the mechanisms of entry may differ when Gh_112 is removed from the highly avid and sterically restricted display system. While this bacterial display-based screening strategy is not likely to identify CPPs capable of direct cell penetration, it selects for protein domains capable of transporting large complex cargo like an entire bacterium across the cell membrane, which may be beneficial for drug delivery platforms.

As we have demonstrated that Gh_112-mediated endocytic uptake, whether the Gh_112 system can deliver cargo outside of the endosome requires further investigation. Recently, several bacterial drug delivery systems have demonstrated the possibility of therapeutic cargo release from within a membrane-bound compartment. An attenuated *Salmonella typhimurium* strain engineered to invade cancerous cells releases oncolytic viral genomes and accessory proteins from within the *Salmonella*-containing vacuole, enabling tumor-selective cell lysis.^15^ Similar work has functionalized nonpathogenic *E. coli* with a recombinant invasin derived from *Yersinia pseudotuberculosis*. These engineered *E. coli* are internalized via the displayed invasin protein and express hemolysin, a protein that naturally permeabilizes the host cell endosome, to release therapeutic cargo to act on cytosolic targets.^40^ Despite the use of nonpathogenic bacterial strains, these systems still leverage intact pathogen-derived proteins for internalization, which could promote immunogenicity. Unlike these systems, the Gh_112 fragment derives from a commensal gut bacterium and is displayed on a nonpathogenic *E. coli* strain, which offers potential advantages with respect to immunogenicity and biosafety. As this work was performed *in vitro*, testing the biodistribution, stability, and immunogenicity of Gh_112 *in vivo* in healthy and tumor models would provide valuable insights into the potential of Gh_112 as a delivery system.

Notably, under conditions tested, Gh_112 preferentially promotes uptake into cancerous epithelial cell lines as compared to healthy, primary cells. This could be due to altered extracellular matrix and receptor expression or rate endocytic flux in immortalized, cancerous cell lines.^41^ We have demonstrated that Gh_112 adheres to human fibronectin and triggers actin rearrangement, two features consistent with fibronectin binding proteins (FnBPs) from *S. aureus*. FnBPs engage with human fibronectin to cluster cell surface integrin receptors, triggering actin rearrangement and bacterial entry.^20^ It is possible that fibronectin acts as the Gh_112 binding partner to mediate internalization; however, further receptor perturbation experiments are required. Interestingly, fibronectin is a glycosylated protein and then becomes more “mucin-like” as cells transition from a healthy to cancerous state.^42^ If Gh_112 interacts with host cells via glycan binding, then this shift in glycosylation pattern could explain the preferential interaction of the protein fragment with cancerous cell lines but not healthy tissue.

In conclusion, we established the human gut microbiome as a source for novel cell-penetrating sequences. As the human gut microbiome harbors thousands of uncharacterized proteins from diverse bacterial species, deeper mining of this community may reveal additional CPPs with varied targeting specificities, cargo capacities, and mechanisms of action. This work expands the available toolbox of cell-penetrating systems and opens new avenues for leveraging the human microbiome in the development of precision-drug-delivery platforms.

## Materials and Methods

### Cell lines

Caco-2 (ATCC HTB-37) and HeLa (ATCC CCL-2) were maintained in Dulbecco’s Modified Eagle Medium (DMEM) + 4.5 g/L glucose without l-glutamine or sodium pyruvate supplemented with 10% fetal bovine serum and used for the internalization assay between passages 5–14. Human colonic epithelial cells (HCoEpiC, ScienCell, 2950) were cultured in Colonic Epithelial Cell Medium (ScienCell, 2951) with 1× penicillin/streptomycin between and used between passages 1–10. Primary cervical epithelial cells (ATCC PCS-480-011) were cultured in Cervical Epithelial Cell Basal Medium (ATCC PCS-480-032) supplemented with Cervical Epithelial Cell Growth Kit (ATCC PCS-480-042). All cell lines were tested for mycoplasma prior to experimentation.

In experiments using differentiated Caco-2, cells were seeded and maintained in DMEM for 14–18 days in flat-bottom tissue-culture-treated plates with medium changed every other day. Caco-2 differentiation was confirmed by expression of the sucrase-isomaltose enzyme as follows: Caco-2 cells were cultured for 2, 7, 14, and 21 days, with three biological replicates per time point. Total RNA was extracted with TRIzol, and complementary DNA (cDNA) was synthesized using the iScript cDNA Synthesis Kit (Bio-Rad), and qPCR was performed with the Luna Universal qPCR Kit (New England Biolabs) for expression of the differentiation marker sucrose-isomaltase while GAPDH served as the internal reference gene.^27^ Gene expression levels were normalized to GAPDH and expression statistics calculated relative to day 2 values.^27^

### Next-Generation Sequencing and Analysis

Human participant research was approved by Cornell University’s Institutional Review Board (IRB) under the protocol number 19-11009200. All participants provided informed consent prior to providing samples. Stool samples were placed in sterile cryovials, frozen immediately after voiding, and stored at −80 °C. Metagenomic sequencing was performed by extraction of total DNA from the fecal samples using the DNeasy PowerSoil Kit (Qiagen) according to the manufacturer’s instructions. Libraries were prepared according to the Nextera XT DNA Library Preparation protocol (Illumina) and sequenced on an Illumina NovaSeq platform using a paired-end 150-cycle run. Reads were dereplicated with PRINSEQ (v0.20.4) with -derep 12345, human-derived sequences were removed using BMTagger, and adapter sequences were trimmed with Trimmomatic v0.39 with ILLUMINACLIP:2:30:10:2:keepBothReads LEADING:3 TRAILING:3 MINLEN:36 to remove adapters ((ILLUMINACLIP:TruSeq3- PE.fa:2:30:10), remove leading low-quality or N bases (below quality 3) (LEADING:3), remove trailing low-quality or N bases (below quality 3) (TRAILING:3), and remove reads below 36 bases long (MINLEN:36). Processed reads from each sample were assembled using SPAdes in metagenomic mode (--meta) (v3.15.5) with k-mer sizes of 21, 29, 39, 59, 79, and 99. The assembled fecal metagenomes were ORF-called with Bakta and annotated for taxonomy with Kraken.^43^

For the surface-displayed libraries, Plasmid DNA was extracted with the Omega E-Z 96 FastFilter Plasmid Kit (Omega Bio-TekD1097-01) according to the manufacturer's instructions on an Eppendorf epMotion 5075 liquid handling robot. Approximately 10 ng of miniprepped DNA was amplified in triplicate with pAIDA-I vector-specific primers with a variable “YRYR” sequence on the forward primer. Triplicates were pooled, amplified with primers containing the Illumina Truseq adapters, and indexed with unique custom 9 bp indices. Libraries were pooled and sequenced on an Illumina MiSeq with a paired-end 150-cycle run and 15% PhiX spike-in.

Reads of the preand postbiopanned display libraries first underwent quality control processing by removal of an Illumina adapter and a vector-specific sequence using CutAdapt. Quality trimming was simultaneously performed with Trimmomatic v0.39 parameters TRAILING:3 MINLEN:25 to preserve the 5′ ends for open reading frame (ORF) calling and remove sequences smaller than 25 bp, and human reads removed with BMTagger.^44,45^ Cleaned reads were mapped with BWA-MEM and filtered with Samtools to keep only mapped (-F 0 × 4), paired (-f 2), primary alignments (-F 2048).^46,47^ Aligned sequences were tallied and used to calculate relative sequence abundance. Read coordinates were then cross-referenced with Bakta-annotated open reading frames and Kraken-assigned taxonomies using custom scripts to identify the source organism and gene from the microbiome and custom scripts used to call inserts forming open reading frames with both the source gene and AIDA1 fusion in the pAIDA1 vector.

### Surface Display Library and Control Construction

Displayed protein sequences were purchased as gBlocks from TWIST. Native signal peptides for the full glycosyl hydrolase enzyme were predicted with SignalP5.0 and removed prior to ordering to allow expression under the AIDA-I signal peptide.^48^ Metagenomic DNA was prepared as previously reported.^49^ Briefly, DNA was extracted from frozen stool, sheared to an average 500 bp fragment size with a Covaris E220 following the manufacturer settings, and end-repaired with the End-It DNA End Repair Kit (Biosearch Technologies, ER81050). The pAIDA-I vector (ATCC #79180) was linearized by inverse PCR with a standard Phusion PCR reaction with the following parameters: 98 °C for 2 min, 25 cycles of: — 98 °C for 30 s — 62 °C for 30 s — 72 °C for 2 min, 72 °C for 10 min. Metagenomic DNA fragments were blunt-ligated into linearized pAIDA-I at a 7:1 molar ratio in a dilute 100 μL reaction with T4 ligase (NEB M0202L) at 16 °C overnight. The ligations were cleaned and transformed into electrocompetent OneshotTop10 (Thermo Fisher C404052) with a Bio-Rad Micropulser, Ec1 setting (pulse 1.80 kV, 5 ms). Transformants were then plated on Luria broth (LB) agar plates with 25 μg/mL chloramphenicol and grown at 37C overnight. After incubation, colonies were scraped down into LB with 20% glycerol and snap frozen in 1 mL aliquots in at least 1000× excess of estimated library size.

Thawed library or control aliquots were grown in LB with 25 μg/mL chloramphenicol to OD600 = 0.2, and then surface-displayed protein expression was induced with 0.2 mM – isopropyl β-d-1-thiogalactopyranoside (IPTG) for 2 h.

### Bacterial Flow Cytometry

Induced cells were pelleted at 3000*g* for 5 min and resuspended in 100 μL of PBS with 1 μL of Alexa Fluor 488 mouse-anti-Myc (Abcam ab202008). Cells were incubated in the dark for 60 min, washed once in PBS, and resuspended in FACS buffer (1× PBS, 0.5% BSA, 2 mM EDTA) with 1 μL of 1 mg/mL propidium iodide to stain for intact membranes. A total of 100,000 events per sample were analyzed on a BD FACSMelody cell sorter and analyzed in FlowJo.

### High-Throughput Functional Metagenomic Internalization Assay

Caco-2 were seeded in six-well plates at a density of 1 × 10^5^ cells/cm^2^ and differentiated as described above. Induced libraries were washed once in PBS, resuspended in DMEM with 1% bovine serum albumin (BSA), and inoculated onto differentiated Caco-2 in six-well plates in 500 μL of DMEM with 1% BSA at a multiplicity of infection (MOI) of 100:1 for the first biopanning round and then 10:1 for subsequent panning rounds. Bacteria were spun down onto plates at 200 × *g* for 10 min and incubated in a humidified, 5% CO_2_, 37 °C incubator for 1 h. Gentamicin was then added to each well at a final concentration of 100 μg/mL and incubated for 90 min. To recover invasive bacteria, media were aspirated from each well and washed once with 1× PBS, and cells were lysed with 300 μL of 0.1% Triton X-100 for 10 min at room temperature followed by addition of 700 μL of LB. 30 μL of recovered lysate or input bacterial library was serially diluted for titering, and remaining lysate was immediately plated on LB agar plates with 25 μg/mL chloramphenicol overnight at 37 °C. Recovered colonies were enumerated, and then plates were scraped down into LB media, incubated for 30 min at 37 °C, and snap-frozen with 20% glycerol for use in subsequent invasion assay rounds. Input library and recovery titers were calculated as follows:

$$\mathrm{CFU}=\frac{\mathrm{average}\;\mathrm{colony}\;\mathrm{count}\;\mathrm{per}\;\mathrm{droplet}\;x\;\mathrm{dilution}\;\mathrm{factor}}{\mathrm{volume}\;\mathrm{of}\;\mathrm{droplet}\;(\mathrm{mL})}\times \mathrm{total}\;\mathrm{sample}\;\mathrm{volume}\;(\mathrm{mL})$$ 1

Relative invasion was calculated as

$$\%\mathrm{CFU}\;\mathrm{recovered}\;\mathrm{of}\;\mathrm{input}=\frac{\mathrm{CFU}\;\mathrm{of}\;\mathrm{post}-\mathrm{gentamicin}\;\mathrm{selected}\;\mathrm{population}}{\mathrm{CFU}\;\mathrm{of}\;\mathrm{input}\;\mathrm{population}}\times 100$$ 2

### Clonal Internalization Assay

Cells were seeded in 96-well plates at the following densities: HeLa (1 × 10^5^ cells/cm^2^), Caco-2 (1 × 10^5^ cells/cm^2^), HCoEpiC (1 × 10^4^ cells/cm^2^), primary cervical epithelial cells (1 × 10^5^ cells/cm^2^). HeLa, HCoEpiC, primary cervical epithelial cells, and undifferentiated Caco-2 were used 24 h postseeding, while differentiated Caco-2 were used 14–18 days postconfluency. The internalization assay was performed as described in Section 4 with several modifications. Clonal-surface display strains were introduced onto mammalian cells at an MOI of 5:1 in 50 μL of mammalian cell culture media. Following gentamicin treatment, cells were lysed in 30 μL of 0.1% Triton X-100 and resuspended in 70 μL of LB. Input and recovered bacteria were enumerated and relative internalization calculated as described in Section 4. Significant differences in invasion were determined by a one-way ANOVA with Dunnett’s multiple comparisons. Samples were considered significant if *p* < 0.05.

For the internalization assay performed with chemical endocytosis inhibitor treatment, the inhibitors or vehicle control were introduced as follows: chlorpromazine HCL (15 μg/mL in H_2_O, Neta Scientific SIAL-C8138), genistein (200 μM in DMSO, VWR TCG0272), dynasore (80 μM in DMSO, Santa Cruz Biotechnology sc-202592), methyl-*B*-cyclodextrin (10 mM in H_2_O, Neta Scientific SIAL-C4555), cytochalasin D (1 μg/mL in DMSO, Life Technologies PHZ1063), amiloride (0.1 mM in DMSO, VWR AAJ62168-03) in DMEM; they were added in approximately 10 min at 37 °C prior to the time of bacterial infection.^25,26^ An equal volume of DMSO or H_2_O vehicle served as control. To quantify the toxicity of the endocytosis inhibitor or vehicle control, the cells were incubated with an inhibitor or vehicle for 10 min at 37 °C with the concentrations indicated above and viability measured by quantifying Trypan blue staining on a Countess2 (Thermo Fisher). For temperature-based inhibition, monolayers were moved to 4 °C at time of inoculation with *E. coli*. Cells were incubated at the described temperature for 1 h. Upon addition of gentamicin, all treatments were incubated at 37 °C for 1.5 h. Relative invasion for each condition was calculated as described in Section 4. Endocytosis inhibition is reported as percent reduction in invasion compared to vehicle control, calculated as follows:

%reductionininternalization=[(%CFUrecoveredofinputinvehiclecontrol−%CFUrecoveredofinputinendocytosisinhibitor)/(%CFUrecoveredofinputinvehiclecontrol)]×100 3

### Bacterial Localization Microscopy

To distinguish intracellular and extracellular *E. coli*, Caco-2 were seeded in ibidi 96-well square coverslip bottom plates (ibidi, 89626) and *E. coli* displaying that Gh_112 or controls were inoculated at an MOI of 100:1 and coincubated with the Caco-2 monolayers for 1 h at 37 °C. Bacteria were differentially stained for intracellular or extracellular localization as previously reported.^18^ Briefly, cells were washed 5× in PBS to remove unbound bacteria. Cells were fixed in 4% methanol-free paraformaldehyde in PBS for 10 min, washed 3× in PBS, and blocked in PBS with 3% bovine serum albumin (BSA) for 1 h and then incubated with 1:200 dilution rabbit anti-*E. coli* (abcam ab13796) in PBS with 1% BSA for 1 h at room temperature. After addition of the primary antibody on unpermeabilized cells, bacteria were stained green by addition of 1:500 Alexa Fluor 488-antirabbit for 1 h at room temperature. The cells were washed 3× to remove unbound secondary and then permeabilized with 0.1% Triton X-100 in PBS for 10 min. The cells were subjected to a second round of staining with the same rabbit anti-*E. coli* antibody, and then the cells were stained with 1:500 Alexa Fluor 647anti-rabbit (Thermo Fisher A-21245). The cells were mounted in Prolong Diamond antifade with DAPI (Invitrogen P36962) and imaged on an Andor/Inverted Olympus IX-83 spinning disc microscope at 100× magnification.

*E. coli* culture was pelleted at 3000 × *g* for 5 min, washed 3× in PBS, and then resuspended in PBS. pHrodo Red succinimidyl ester (Thermo Fisher P36600) was resuspended in fresh anhydrous DMSO according to the manufacturer’s instructions. 5 μL of resuspended dye stock was mixed with each *E. coli* suspension and incubated rotating in the dark at room temperature for 60 min. Bacteria were washed 5× in PBS to remove free dye, resuspended in DMEM, and inoculated onto HeLa seeded as described in Section 5 on ibidi 96-well square-cover glass-bottom plates at an MOI of 1:100. After allowing 3 h for invasion at 37 °C, CellMask Green Actin Tracking stain (Life Technologies A57243) was prepared according to the manufacturer’s instructions and 2 μL was added to each well. Cells were incubated for an additional hour at 37 °C. DMEM was removed and replaced with OptiMEM (Life Technologies 31985070) for live cell imaging under a Zeiss 710 inverted confocal microscope.

### Fibronectin Binding Assay

Induced *E. coli* display cultures showing indicated proteins were washed 3× and resuspended in PBS. SYBR-Gold nucleic acid stain (Life Technologies S11494) was added to each *E. coli* suspension at a 1× final concentration and incubated in the dark for 60 min rotating at room temperature to label bacteria. Bacteria were washed 5× in PBS and introduced into a 96-well fibronectin-coated plate (Neta Scientific MILL-ECM101) in a 1 × 10^7^ bacterial well in 0.1 mL in technical triplicate. Bacteria were spun down onto the plates at 3000 × *g* for 10 min and incubated at 37 °C for 1 h. The wells were washed 5× to remove unbound bacteria and read on a Cytation 5 plate reader at ex485/em528. Images of each well were taken on a Leica DMi8 inverted LED fluorescence light microscope.

### Gh_112 Protein Analysis

Protein properties (molecular weight and isoelectric point) were estimated with the Expasy ProtParam tool. Predicted cell-penetrating peptides within the Gh_112 sequence were called with the CellPPD “protein scanning” tool with the parameters SVM = 0 and were queried at all possible k-mer lengths of 10, 15, 20, 25, 30, 35, 40, 45, and 50, and known CPPs were identified by the “peptide mapping” feature on the CPPSite3.0 Web server.

To identify all homologous sequences and structures to Gh_112, we performed a standard BLAST search on the NCBI BLASTp server and queried the AlphaFold structure on the DALI protein search server. Gh_112 was scanned for binding motifs with Pfam and manual search for the known integrin-binding sequence “RGD”. To identify homologous fragments from human microbiomes, we performed a custom BLASTp search against the curatedMetagenomicData data set. We then queried the custom database using diamond BLASTp with the parameters (--evalue 0.0000000001). Unrooted maximum likelihood phylogenetic trees were constructed using SEAVIEW with PHML, LG model with 100 bootstraps.^50^ Multiple sequence alignments were made with ClustalOmega and colored in Jalview according to amino acid family.^51,52^

## Supplementary Material



## Notes

Supplementary figure file including The Supporting Information is available free of charge at https://pubs.acs.org/doi/10.1021/acssynbio.6c00031.

(S1) Metagenomic display library size and length distribution; (S2) diagrams of the pAIDA1 display system; (S3) sucrase-isomaltase differentiation marker expression of Caco-2; (S4) flow cytometry gating strategy for surface-localized Myc tag detection; (S5) gentamicin sensitivity testing for surface-displayed constructs; (S6) pAIDA1-v2 vector diagram and pAIDA1-v2 display internalization assay; (S7) flow-cytometry quantified surface display efficiency of Gh_112 halves; and (S8) Caco-2 viability after endocytosis inhibitor treatment (PDF)
