# Supplementary material for: Discovery of Novel Glycosidase-Derived Cell-Penetrating Peptides Encoded by Human Gut Commensals
Source: ACS Synth Biol. 2026 Jul 9;15(8):3179–92. doi: 10.1021/acssynbio.6c00031 (PMC13505336; doi:10.1021/acssynbio.6c00031)
Supplement: Supplementary file 1 [file sb6c00031_si_001.pdf]

Discovery of novel glycosidase-derived cell-penetrating peptides encoded by human gut commensals

Sarah E. Post, Hannah S. Ceisler, Rohit G. Lal, Adarsh Singh, Maya A. Deen, Locke E. Bonomo, Lille M. Cunic, Ilana L. Brito

Affiliations

Meinig School for Biomedical Engineering, Cornell University, Ithaca, NY, United States of America

* Please send all correspondences: to ibrito@cornell.edu

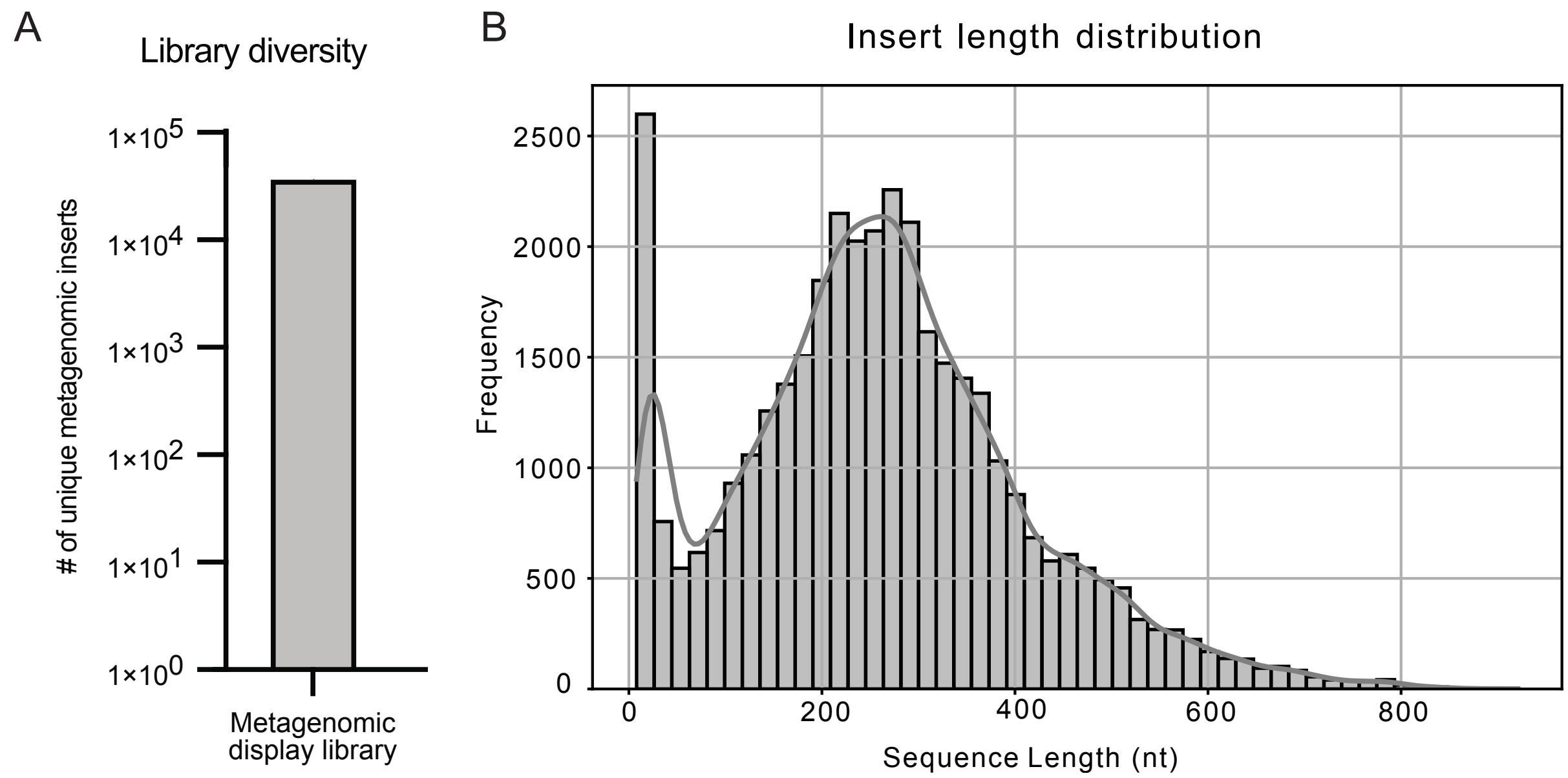

Supplementary Figure 1. Metagenomic display library construction metrics

(A) Number of unique metagenomic inserts present and **(B)** distribution of metagenomic fragment lengths in the display library

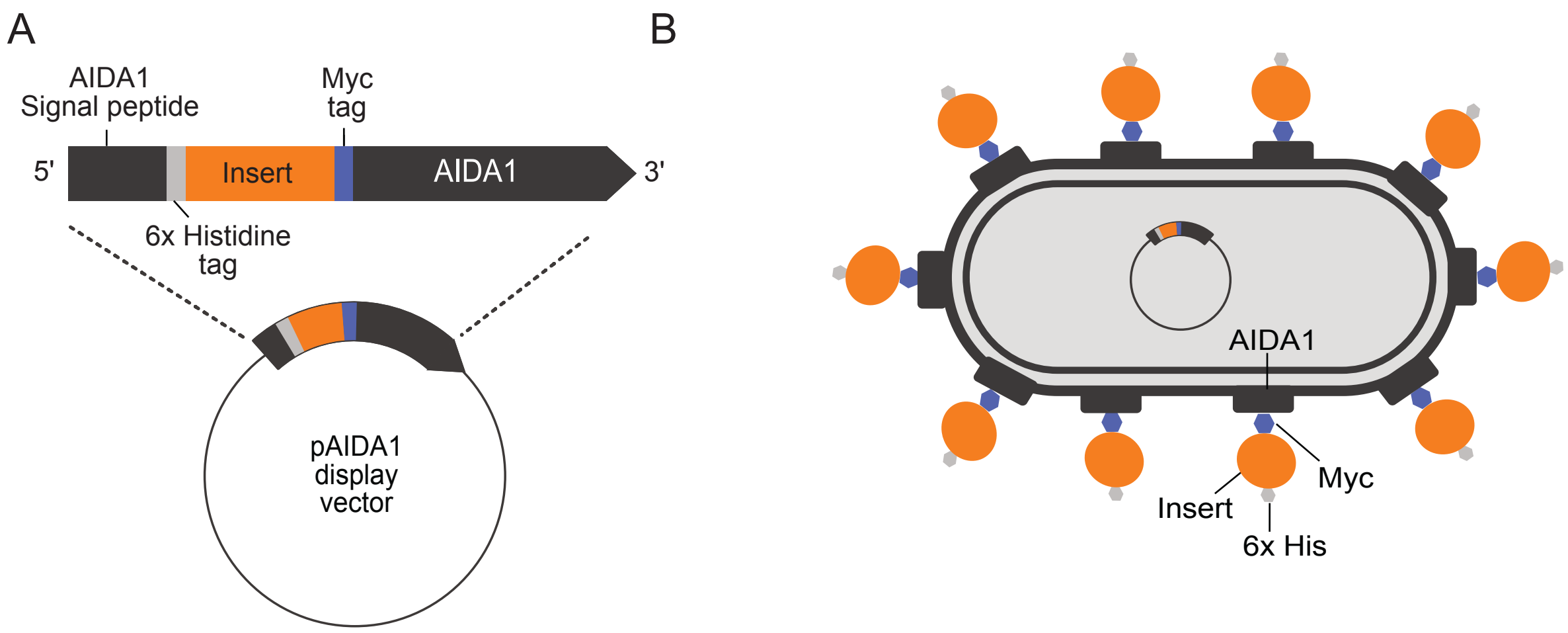

Supplementary Figure 2. Overview of the pAIDA1 surface display system

(A) Diagram of the AIDA1 surface display expression system in the pAIDA1 vector **(B) T**he AIDA1 expression construct as displayed on the *E. coli* cell surface

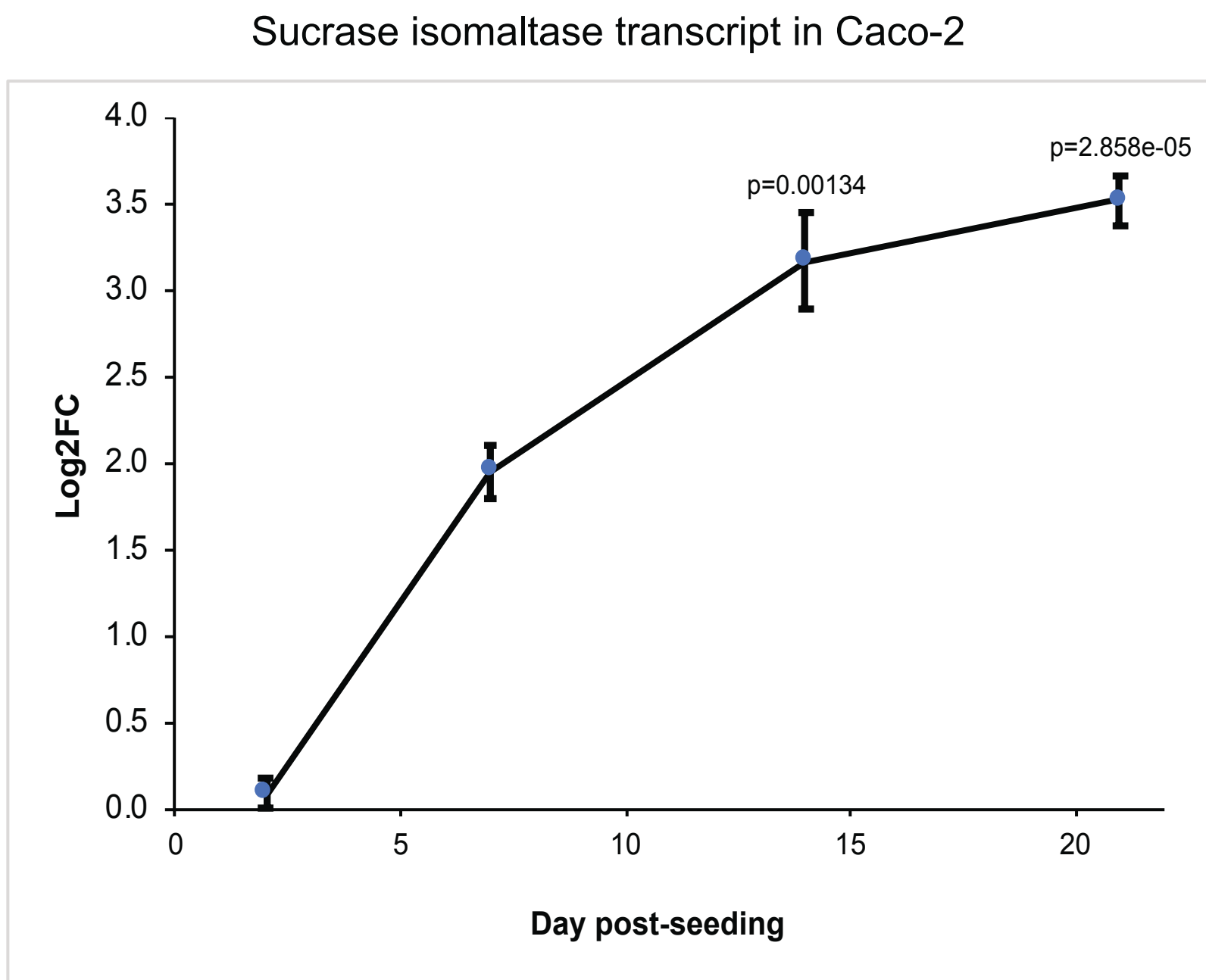

Supplementary Figure 3. Sucrase-isomaltase expression as indicator of Caco-2 differentiation status.

Relative expression of sucrase-isomaltase transcript in Caco-2 cultured continuously from day 2 to day 21 post-seeding normalized to the housekeeping gene GAPDH. p-value calculated by student T-test compared to day 2 samples.

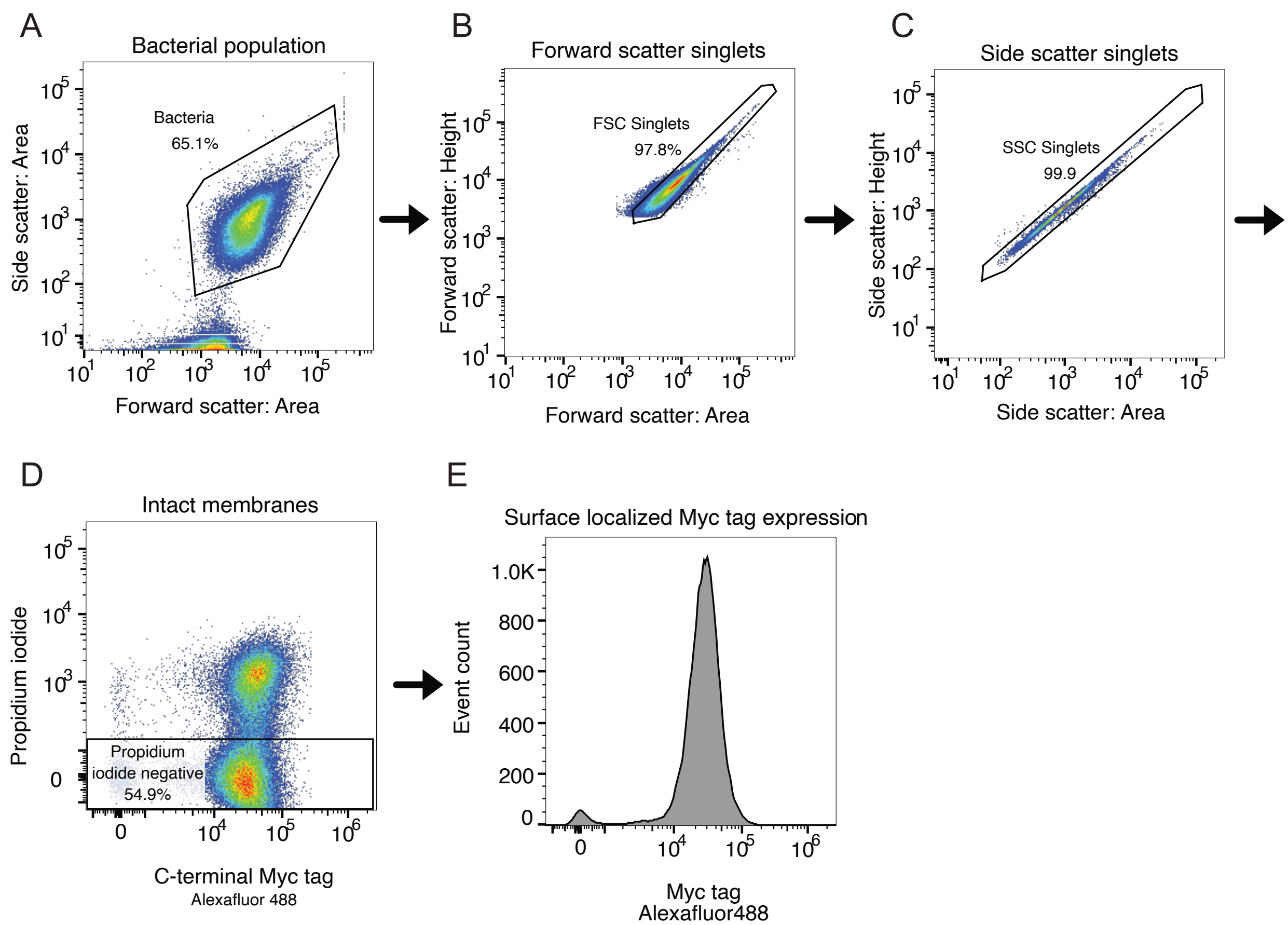

Supplementary Figure 4. Gating strategy for surface-localized Myc tag expression.

Identification of surface displayed protein expression on *E. coli* determined by sequential gating for **(A)** bacteria **(B)** single bacteria (forward scatter singlets) **(C)** single bacteria (side scatter singlets) **(D)** intact membranes (propidium iodide negative) **(E)** Myc tag expression.

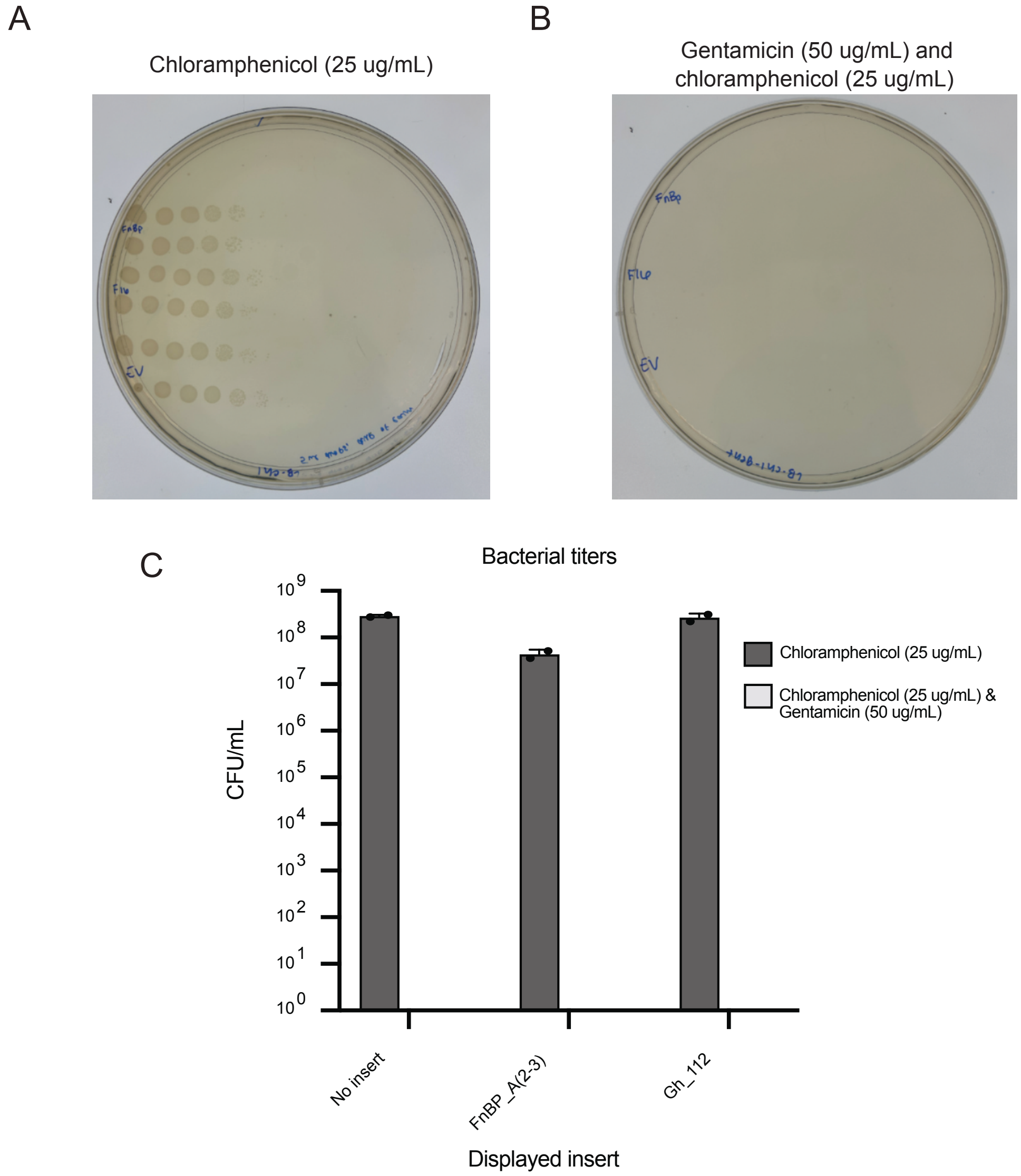

Supplementary Figure 5. Gh_112 is gentamicin sensitive.

Growth of *E. coli* displaying Gh_112 or controls on **(A)** LB - 25 ug/ml chloramphenicol plates versus **(B)** 100ug/mL LB- gentamicin +25ug/mL chloramphenicol plates after protein induction. Plates contain 0.2mM IPTG to continuously induce protein display during overnight culture at 37C. Colony titers quantified in (**C**).

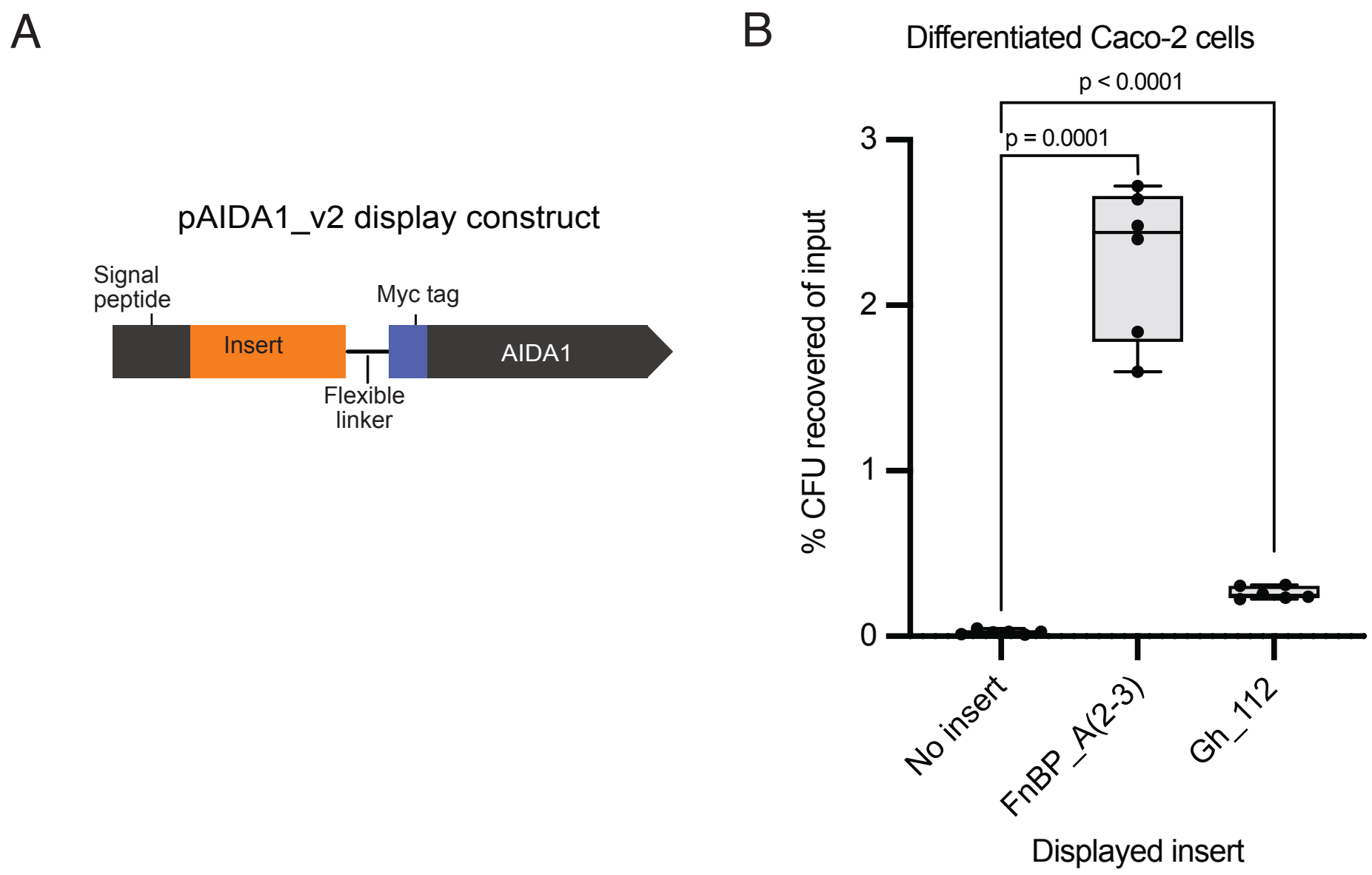

Supplementary Figure 6. Internalization of Gh_112 -displaying bacteria from the pAIDA_v2 vector without histidine residues.

(A) Diagram of the AIDA1 surface display expression system in the pAIDA1_v2 vector. **(B)** Relative recovery of *E. coli* displaying no insert, FnBP_A(2-3), or Gh_112 from the pAIDA1_v2 vector following the internalization assay in differentiated Caco-2. (n=3). Error bars reflect standard deviation, p-values < 0.05 indicated as measured via One-Way ANOVA with post-hoc Dunnett's multiple comparisons against the 'no insert' control.

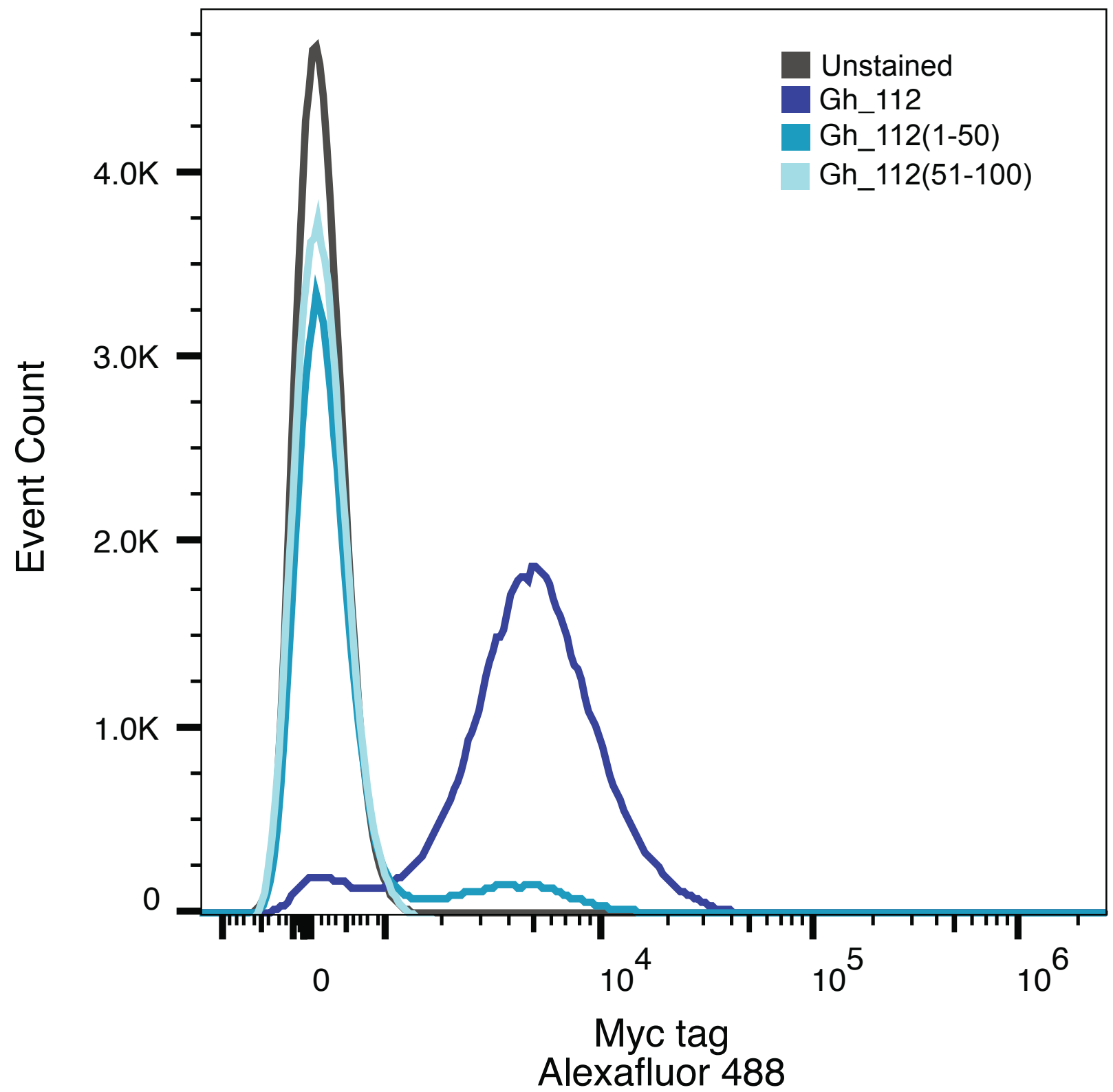

Supplementary Figure 7. Display efficiency of Gh_112 halves on *E. coli* surface.

Surface localization of the 50- amino acid halves of Gh_112, Gh_112(1-50) and Gh_112(51-100) on the surface of *E. coli* as compared to unstained control and the 100-amino acid Gh_112 fragment measured by detection of the Myc tag on propidium-iodide negative (intact membrane) bacterial cell population via flow cytometry.

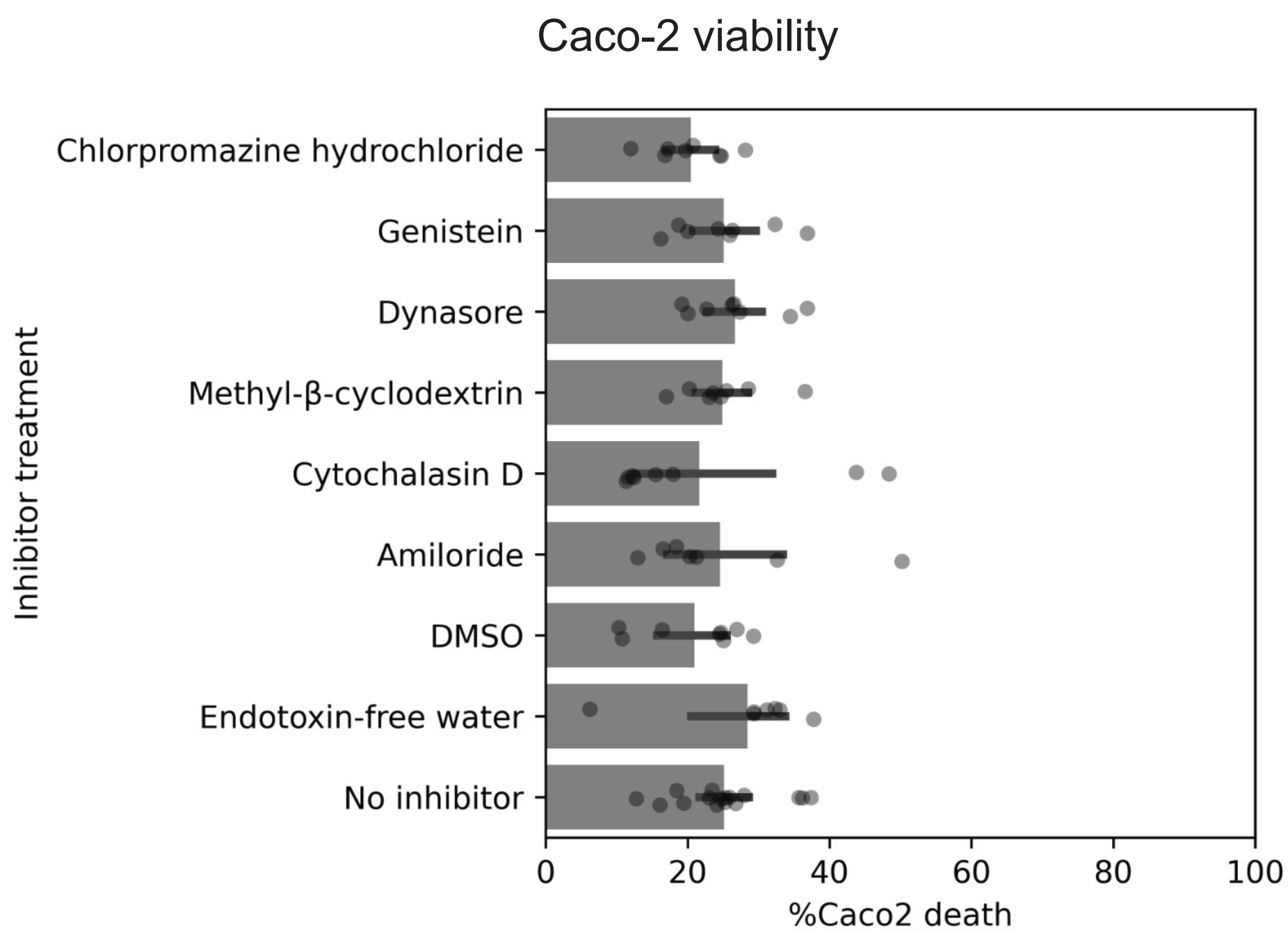

Supplementary Figure 8. Cell viability following endocytosis inhibitor treatment.

Caco-2 viability following treatment with endocytosis inhibitor or vehicle for 10 minutes at 37°C as measured by Trypan blue staining.
